# Prevalence of *Cryptosporidium*, microsporidia and *Isospora* infection in HIV-infected people: a global systematic review and meta-analysis

**DOI:** 10.1186/s13071-017-2558-x

**Published:** 2018-01-09

**Authors:** Ze-Dong Wang, Quan Liu, Huan-Huan Liu, Shuang Li, Li Zhang, Yong-Kun Zhao, Xing-Quan Zhu

**Affiliations:** 1 State Key Laboratory of Veterinary Etiological Biology, Key Laboratory of Veterinary Parasitology of Gansu Province, Lanzhou Veterinary Research Institute, Chinese Academy of Agricultural Sciences, Lanzhou, Gansu Province 730046 People’s Republic of China; 2 Military Veterinary Institute, Key Laboratory of Jilin Province for Zoonosis Prevention and Control, Academy of Military Medical Sciences, Changchun, Jilin Province 130122 People’s Republic of China; 3 College of Animal Science and Technology, Jilin Agricultural University, Changchun, Jilin Province 130188 People’s Republic of China

**Keywords:** HIV, *Cryptosporidium*, Microsporidia, *Isospora*, Meta-analysis

## Abstract

**Background:**

Diarrhea caused by opportunistic intestinal protozoa is a common problem in HIV infection. We aimed to establish the prevalence of *Cryptosporidium*, misrosporidia, and *Isospora* in HIV-infected people using a systematic review and meta-analysis, which is central to developing public policy and clinical services.

**Methods:**

We searched PubMed, ScienceDirect, Google Scholar, Embase, Chinese Web of Knowledge, Wanfang, and Chongqing VIP databases for studies reporting *Cryptosporidium*, microsporidia, or *Isospora* infection in HIV-infected people. We extracted the numbers of people with HIV and protozoa infection, and estimated the pooled prevalence of parasite infection by a random effects model.

**Results:**

Our research identified 131 studies that reported *Cryptosporidium*, microsporidia, and *Isospora* infection in HIV-infected people. We estimated the pooled prevalence to be 14.0% (3283/43,218; 95% CI: 13.0–15.0%) for *Cryptosporidium*, 11.8% (1090/18,006; 95% CI: 10.1–13.4%) for microsporidia, and 2.5% (788/105,922; 95% CI: 2.1–2.9%) for *Isospora*. A low prevalence of microsporidia and *Isospora* infection was found in high-income countries, and a high prevalence of *Cryptosporidium* and *Isospora* infection was found in sub-Saharan Africa. We also detected a high prevalence of *Cryptosporidium*, microsporidia, and *Isospora* infection in patients with diarrhea. Sensitivity analysis showed that three studies significantly affect the prevalence of *Isospora*, which was adjusted to 5.0% (469/8570; 95% CI: 4.1–5.9%) by excluding these studies.

**Conclusions:**

Our findings suggest that HIV-infected people have a high prevalence of *Cryptosporidium*, microsporidia, and *Isospora* infection in low-income countries and patients with diarrhea, especially in sub-Saharan Africa, reinforcing the importance of routine surveillance for opportunistic intestinal protozoa in HIV-infected people.

**Electronic supplementary material:**

The online version of this article (10.1186/s13071-017-2558-x) contains supplementary material, which is available to authorized users.

## Background

Despite the advance of antiretroviral therapy (ART), diarrhea is still a common problem of HIV infection and contributes to the reduced life quality and survival of HIV patients [1, 2]. It is estimated that diarrhea occurs in roughly 90% HIV/AIDS patients in developing countries, and 30–60% in developed countries [3]. Opportunistic pathogens that cause diarrhea in HIV-infected people include protozoa, fungi, viruses, and bacteria [4]. Several protozoan species belonging to *Cryptosporidium*, microsporidia and *Isospora*, are among the most common causative pathogens responsible for significant morbidity and mortality in HIV patients [5].

With a worldwide distribution of *Cryptosporidium*, *C. parvum* and *C. hominis* are the most common species detected in humans, though other species, including *C. meleagridis*, *C. felis* and *C. canis*, have also been reported [6]. Despite the use of ART in many countries of the world, the infection rates of *Cryptosporidium* in HIV patients are still high, accounting for up to a third of diarrhea cases in HIV patients [7].

Microsporidia are obligate intracellular eukaryotic pathogens, which are phylogenetically related to fungi, and have been considered as opportunistic infections in both developed and developing countries, especially in HIV patients with a CD4 cell count below 100 cells/μl [8]. Of the 15 species of microsporidia that infect humans, *Enterocytozoon bieneusi* and *Encephalitozoon intestinalis* can cause gastrointestinal diseases, with *E. bieneusi* being the more commonly identified species in HIV-infected people [9].

*Isospora belli* is the only species of the genus *Isospora*, and is frequently found in HIV-infected people of tropical and subtropical regions, accounting for up to 20% of diarrhea cases in AIDS patients [7]. The species can cause acute self-limiting diarrhea in immunocompetent individuals, but in severely immunocompromised patients, this parasite can cause severe chronic diarrhea which may result in a wasting syndrome, or even the death of AIDS patients [10].

The opportunistic parasites *Cryptosporidium* spp., microsporidians and *Isospora* spp. develop in enterocytes, and are excreted via feces and transmitted through the fecal-oral route via ingestion of contaminated water or food, or direct contact with infected animals or humans [11]. HIV-infected people are more likely to develop abrupt, severe, and explosive diarrhea when infected with opportunistic protozoa than immunocompetent individuals. Millions of people are affected by the morbidity caused by these parasites, as there was an estimated 36.7 million people living with HIV in 2015 worldwide [12]. Since there is no reliable or well-defined treatment for the protozoan infections in immunocompromised patients [1], understanding their epidemiology is central in formulating effective control strategies against cryptosporidiosis, microsporidiosis, and isosporiasis in these populations. We undertook a systematic review and meta-analysis to evaluate the worldwide prevalence of *Cryptosporidium*, microsporidia and *Isospora* infection in people with HIV.

## Methods

### Search strategy

We searched PubMed, ScienceDirect, Google Scholar, Embase, Chinese Web of Knowledge, Wanfang, and Chongqing VIP databases for studies reporting *Cryptosporidium*, microsporidia, or *Isospora* infection in HIV-infected people from inception to 31 December 2016. The databases were searched using the term “*Cryptosporidium*”, “cryptosporidiosis”, “microsporidia”, “microsporidiosis”, “*Isospora*” or “isosporiasis” cross-referenced with “HIV”, “immunodeficiency”, “acquired immune deficiency syndrome”, or “AIDS”, without language restriction. We did our analyses according to the Preferred Reporting Items for Systematic Reviews and Meta-Analyses (PRISMA) statement [13] (see PRISMA checklist in Additional file 1: Table S1).

### Selection criteria

The included studies were required to investigate HIV-infected people and needed to have data that allowed us to calculate the prevalence of *Cryptosporidium*, microsporidia, and *Isospora* infection. We excluded studies if they were reviews, animal studies, or repeated studies; if there were no raw data; if the sample size was less than 20; or if the diagnostic methods of parasite infection were unclear.

Two independent reviewers (LZ and SL) carefully examined all titles and abstracts identified in the search, and assessed the full text considered potentially relevant. Any disagreements were resolved by discussion with other two authors (Z-DW and H-HL).

### Data analysis

Two reviewers (Z-DW and SL) extracted the information about the first author, publication year, country of the study, numbers of HIV-infected people and *Cryptosporidium*, microsporidia, or *Isospora* co-infected people, diagnostic methods, study design, and demographic characteristics from each eligible study, and reached a consensus after discussing any controversial finding.

We assessed the quality of the included publications on the basis of criteria derived from the Grading of Recommendations Assessment, Development and Evaluation method [14]. We used a scoring approach to grade quality. Studies were given one point each if they had probability sampling, larger sample sizes of more than 200, and repeated detection. Up to four points could be assigned to each study. We regarded publications with a total score of three or four points to be of high quality, whereas two points represented moderate quality and scores of one or zero represented low quality.

We did a meta-analysis by a random-effects model or fixed-effects model to calculate the pooled prevalence of *Cryptosporidium*, microsporidia, or *Isospora* infection using Stata version 12.

The heterogeneity between studies was evaluated using Cochran’s Q and the *I*^2^-statistic, which presents the percentage of variation between studies. Due to high heterogeneity (*I*^2^ > 50%, *P* < 0.1), random effects models were used for summary statistics. A potential source of heterogeneity was investigated by subgroup analysis and meta-regression analysis. We examined factors both individually and in multiple-variable models to determine the possible factors that caused heterogeneity in our study. The factors included geographical region by comparison of sub-Sahara Africa with other regions, income level by comparison of low-income countries with others, and patients with diarrhea by comparison of patients with diarrhea with others. We also evaluated the effect of selected studies on the pooled prevalence by excluding single studies sequentially. A study was considered to have no influence if the pooled estimate without it was within the 95% confidence limits of the overall prevalence [15].

## Results

**Fig. 1:**
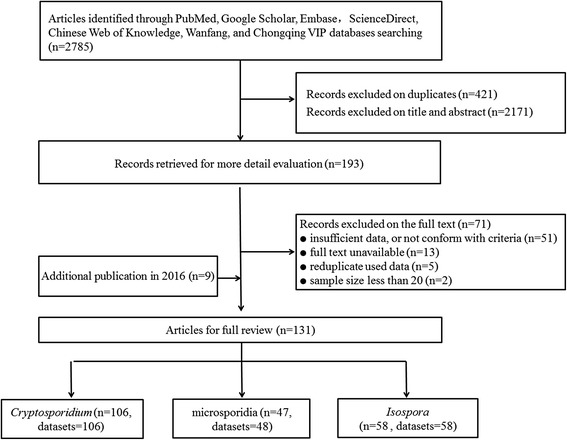
Flowchart of the study selection process

Our research identified 2785 records. After initial screening and removal of duplicates, 193 papers were reviewed in full. Of these, 51 articles did not include sufficient data that were required or conform to the criteria, 13 were unavailable for full text, five had duplicate samples, and two included the sample size of less than 20. After an updated search, nine papers were included and we had 131 articles for quality assessment and meta-analysis (Fig. 1).

According to our criteria, 51 publications were of high quality with a score of three or four, 48 had a score of two indicating moderate quality, and the remaining 32 were of low quality with a score of zero or one (Tables 1, 2 and 3).

**Table 1:** Included studies of *Cryptosporidium* infection in people with HIV listed in order of year published

	Country	Income level	Patients with diarrhea	No. of patients	Prevalence (%)	Quality score
Western and central Europe and North America						
René et al. (1989) [37]	France	High	Mixed	132	21.2	2
Connolly et al. (1990) [53]	UK	High	Yes	33	15.2	1
Brandonisio et al. (1993) [54]	Italy	High	Yes	51	33.3	1
Sorvillo et al. (1994) [41]	USA	High	Mixed	16,953	3.8	2
Colford et al. (1996) [34]	USA	High	Mixed	3564	5.4	3
Mathewson et al. (1998) [42]	USA	High	Yes	83	10.8	2
Matos et al. (1998) [35]	Portugal	High	Yes	465	7.7	3
Brandonisio et al. (1999) [38]	Italy	High	Mixed	154	11.0	3
Cama et al. (2006) [55]	USA	High	Mixed	21	33.3	1
Lagrange-Xelot et al. (2008) [27]	France	High	Mixed	6827	1.3	1
Sub-Saharan Africa						
Henry et al. (1986) [32]	DR Congo	Low	Yes	46	8.7	0
Colebunders et al. (1988) [56]	DR Congo	Low	Yes	42	31.0	0
Therizol-Ferly et al. (1989) [57]	Ivory Coast	Middle	Yes	148	6.8	1
Hunter et al. (1992) [58]	Zambia	Middle	Mixed	90	2.2	2
Assoumou et al. (1993) [59]	Ivory Coast	Middle	Mixed	217	8.8	1
Dieng et al. (1994) [60]	Senegal	Low	Yes	72	13.9	1
Chintu et al. (1995) [61]	Zambia	Middle	Yes	44	13.6	2
Mwachari et al. (1998) [62]	Kenya	Middle	Yes	75	17.3	2
Fisseha et al. (1999) [63]	Ethiopia	Low	Mixed	190	20.0	2
Gumbo et al. (1999) [31]	Zimbabwe	Low	Yes	82	8.5	2
Cegielski et al. (1999) [64]	Tanzania	Low	Yes	86	7.0	2
Lebbad et al. (2001) [65]	Guinea-Bissau	Low	Yes	37	21.6	2
Nwokediuko et al. (2002) [66]	Nigeria	Middle	Yes	161	0.0	1
Adjei et al. (2003) [67]	Ghana	Middle	Yes	21	28.6	2
Tumwine et al. (2005) [28]	Uganda	Low	Yes	91	73.6	2
Tadesse et al. (2005) [68]	Ethiopia	Low	Yes	70	28.6	1
Sarfati et al. (2006) [69]	Cameroon	Middle	Mixed	154	9.7	3
Adesiji et al. (2007) [29]	Nigeria	Middle	Yes	100	79.0	3
Mariam et al. (2008) [70]	Ethiopia	Low	Mixed	109	7.3	2
Blanco et al. (2009) [71]	Equatorial Guinea	Middle	na	171	18.1	3
Cooke et al. (2009) [72]	South Africa	Middle	Mixed	26	7.7	0
Babatunde et al. (2010) [73]	Nigeria	Middle	Mixed	90	32.2	1
Alemu et al. (2011) [74]	Ethiopia	Low	Mixed	188	43.6	1
Bartelt et al. (2011) [30]	South Africa	Middle	na	193	75.6	1
Roka et al. (2012) [75]	Equatorial Guinea	Middle	Mixed	260	9.2	4
Wumba et al. (2012) [76]	DR Congo	Low	Mixed	242	5.4	4
Nwuba et al. (2012) [33]	Nigeria	Middle	Mixed	202	30.7	3
Girma et al. (2014) [77]	Ethiopia	Low	Mixed	268	34.3	3
Samie et al. (2014) [78]	South Africa	Middle	Mixed	151	26.5	2
Vouking et al. (2014) [79]	Cameroon	Middle	Mixed	207	7.2	3
Bissong et al. (2015) [80]	Cameroon	Middle	Mixed	200	7.0	3
Kiros et al. (2015) [81]	Ethiopia	Low	Mixed	399	5.8	3
Nsagha et al. (2016) [39]	Cameroon	Middle	Mixed	300	44.0	4
Shimelis et al. (2016) [3]	Ethiopia	Low	Mixed	491	13.2	3
Ojuromi et al. (2016) [82]	Nigeria	Middle	Mixed	90	4.4	3
Asia and the Pacific						
Kamel et al. (1994) [83]	Malaysia	Middle	Mixed	100	23.0	0
Moolasart et al. (1995) [84]	Thailand	Middle	Yes	250	8.8	2
Anand et al. (1996) [85]	India	Middle	Mixed	200	35.0	1
Punpoowong et al. (1998) [86]	Thailand	Middle	Yes	22	9.1	0
Wanachiwanawin et al. (1999) [87]	Thailand	Middle	Yes	91	25.3	2
Prasad et al. (2000) [88]	India	Middle	Mixed	26	11.5	2
Wiwanitkit et al. (2001) [89]	Thailand	Middle	Mixed	60	3.3	1
Chokephaibulkit et al. (2001) [90]	Thailand	Middle	Yes	82	6.1	2
Waywa et al. (2001) [91]	Thailand	Middle	Yes	288	19.1	3
Kumar et al. (2002) [92]	India	Middle	Mixed	100	14.0	2
Mohandas et al. (2002) [93]	India	Middle	Mixed	120	10.8	3
Lim et al. (2005) [94]	Malaysia	Middle	Mixed	66	3.0	1
Guk et al. (2005) [95]	South Korea	High	Mixed	67	10.4	1
Chhin et al. (2006) [96]	Cambodia	Middle	Yes	80	45.0	3
Dwivedi (2007) [48]	India	Middle	Mixed	75	33.3	2
Ramakrishnan et al. (2007) [97]	India	Middle	Yes	80	28.8	2
Qu et al. (2007) [19]	China	Middle	Yes	141	3.5	0
Stark et al. (2007) [98]	Australia	High	Yes	618	2.3	4
Saldanha et al. (2008) [99]	India	Middle	na	307	17.3	1
Jayalakshmi et al. (2008) [43]	India	Middle	Yes	89	12.4	2
Viriyavejakul et al. (2009) [100]	Thailand	Middle	Mixed	64	20.3	2
Saksirisampant et al. (2009) [101]	Thailand	Middle	Mixed	90	34.4	1
Kulkarni et al. (2009) [44]	India	Middle	Yes	137	11.7	1
Guo et al. (2011) [20]	China	Middle	Yes	149	16.1	2
Tian et al. (2012) [102]	China	Middle	na	302	8.3	4
Tian et al. (2012) [22]	China	Middle	Mixed	46	13.0	3
Li et al. (2012) [21]	China	Middle	Yes	67	6.0	2
Wang et al. (2012) [23]	China	Middle	Yes	253	12.6	3
Sherchan et al. (2012) [103]	Nepal	Low	Mixed	146	2.7	3
Wang et al. (2013) [9]	China	Middle	Mixed	673	1.5	4
Mehta et al. (2013) [104]	India	Middle	Mixed	100	2.0	3
Vyas et al. (2013) [105]	India	Middle	Yes	75	14.7	2
Gupta et al. (2013) [45]	India	Middle	Mixed	100	4.0	2
Baragundi Mahesh et al. (2013) [106]	India	Middle	Mixed	75	18.7	2
Paboriboune et al. (2014) [107]	Laos	Middle	Mixed	137	6.6	3
Jain et al. (2014) [108]	India	Middle	Mixed	250	20.8	2
Pang et al. (2015) [16]	China	Middle	na	450	17.3	3
Angal et al. (2015) [109]	Malaysia	Middle	Mixed	131	3.8	3
Xie et al. (2015) [17]	China	Middle	Mixed	152	13.2	0
Khalil et al. (2015) [110]	India	Middle	Mixed	200	7.5	3
Asma et al. (2015) [111]	Malaysia	Middle	Mixed	346	12.4	4
Kaniyarakkal et al. (2016) [112]	India	Middle	Mixed	200	2.5	2
Mitra et al. (2016) [113]	India	Middle	Mixed	194	29.4	2
Shah et al. (2016) [114]	India	Middle	Mixed	45	13.3	2
Wang et al. (2016) [18]	China	Middle	Mixed	285	0.7	4
Latin America and the Caribbean						
Chacin-Bonilla et al. (1992) [115]	Venezuela	High	Mixed	29	41.4	1
Escobedo et al. (1999) [116]	Cuba	Middle	Mixed	67	11.9	2
Florez et al. (2003) [117]	Colombia	Middle	Mixed	115	10.4	3
Ribeiro et al. (2004) [118]	Brazil	Middle	Mixed	75	9.3	2
Chacin et al. (2006) [119]	Venezuela	High	Yes	103	25.2	2
Goncalves et al. (2009) [120]	Brazil	Middle	Mixed	100	9.0	2
Cardoso et al. (2011) [121]	Brazil	Middle	Mixed	500	0.2	3
Velasco et al. (2011) [122]	Colombia	Middle	Mixed	131	29.0	2
Guimarães et al. (2012) [123]	Brazil	Middle	Mixed	93	2.2	1
Assis et al. (2013) [124]	Brazil	Middle	Mixed	59	10.2	2
Middle East and North Africa						
Zali et al. (2004) [125]	Iran	Middle	Mixed	206	1.5	2
Yosefi et al. (2012) [126]	Iran	Middle	Mixed	60	8.3	2
Agholi et al. (2013) [127]	Iran	Middle	Mixed	356	9.6	3
Salehi Sangani et al. (2016) [128]	Iran	Middle	Mixed	80	1.3	2
Eastern Europe and central Asia						
Brannan et al. (1996) [129]	Romania	Middle	Mixed	73	78.1	3
Kucervoa et al. (2011) [130]	Russia	Middle	na	46	41.3	2

*Abbreviations*: Yes, patients with diarrhea; Mixed, including patients with or without diarrhea; na, not applicable (parameter not provided)

**Table 2:** Included studies of microsporidia infection in people with HIV listed in order of year published

	Country	Income level	Patients with diarrhea	No. of patients	Prevalence (%)	Quality score
Western and central Europe and North America						
Weber et al. (1992) [131]	USA	High	Mixed	134	4.5	2
Kotler et al. (1994) [132]	USA	High	Mixed	194	28.9	3
Anwar-Bruni et al. (1996) [36]	USA	High	Mixed	371	5.9	4
Coyle et al. (1996) [133]	USA	High	Mixed	111	27.9	3
Mathewson et al. (1998) [42]	USA	High	Yes	83	6.0	2
Brandonisio et al. (1999) [38]	Italy	High	Mixed	154	4.5	3
Ferreira et al. (2001) [134]	Portugal	High	Yes	215	42..8	4
Lagrange-Xelot et al. (2008) [27]	France	High	Mixed	6827	0.8	1
Sub-Saharan Africa						
van Gool et al. (1995) [135]	Zimbabwe	Low	Yes	129	10.1	2
Maiga et al. (1997) [24]	Mali	Low	Mixed	77	32.5	1
Mwachari et al. (1998) [62]	Kenya	Middle	Yes	36	2.8	2
Cegielski et al. (1999) [64]	Tanzania	Low	Yes	86	3.5	2
Gumbo et al. (1999) [31]	Zimbabwe	Low	Yes	55	50.9	2
Lebbad et al. (2001) [65]	Guinea-Bissau	Low	Yes	37	8.1	2
Endeshaw et al. (2005) [136]	Ethiopia	Low	Yes	80	22.5	1
Tumwine et al. (2005) [28]	Uganda	Low	Yes	91	76.9	2
Endeshaw et al. (2006) [137]	Ethiopia	Low	Yes	214	18.2	3
Sarfati et al. (2006) [69]	Cameroon	Middle	Mixed	154	5.2	3
Breton et al. (2007) [138]	Gabon	Middle	na	822	3.0	4
Breton et al. (2007) [138]	Cameroon	Middle	na	758	2.9	4
Akinbo et al. (2012) [139]	Nigeria	Middle	Mixed	463	16.6	3
Wumba et al. (2012) [76]	DR Congo	Low	Mixed	242	8.3	4
Bissong et al. (2015) [80]	Cameroon	Middle	Mixed	200	2.0	3
Nsagha et al. (2016) [39]	Cameroon	Middle	Mixed	300	21.3	4
Ojuromi et al. (2016) [82]	Nigeria	Middle	Mixed	90	5.6	3
Asia and the Pacific						
Punpoowong et al. (1998) [86]	Thailand	Middle	Yes	22	27.3	0
Wanachiwanawin et al. (1998) [140]	Thailand	Middle	Yes	66	33.3	3
Wanachiwanawin et al. (1999) [87]	Thailand	Middle	Yes	91	28.6	2
Chokephaibulkit et al. (2001) [90]	Thailand	Middle	Yes	82	19.5	2
Wiwanitkit et al. (2001) [89]	Thailand	Middle	Mixed	60	1.7	1
Waywa et al. (2001) [91]	Thailand	Middle	Yes	288	9.7	3
Kumar et al. (2002) [92]	India	Middle	Mixed	150	0.7	2
Wanachiwanawin et al. (2002) [141]	Thailand	Middle	Yes	95	25.3	2
Mohandas et al. (2002) [93]	India	Middle	Mixed	120	2.5	3
Dwivedi et al. (2007) [48]	India	Middle	Mixed	75	6..7	2
Saksirisampant et al. (2009) [101]	Thailand	Middle	Mixed	90	5.6	1
Viriyavejakul et al. (2009) [100]	Thailand	Middle	Mixed	64	81.3	2
Kulkarni et al. (2009) [44]	India	Middle	Yes	137	1.5	1
Wang et al. (2013) [9]	China	Middle	Mixed	683	5.7	4
Xie et al. (2015) [17]	China	Middle	Mixed	152	5.3	0
Khalil et al. (2015) [110]	India	Middle	Mixed	200	2.5	3
Khanduja et al. (2016) [8]	India	Middle	Mixed	222	1.8	4
Mitra et al. (2016) [113]	India	Middle	Mixed	194	2.1	2
Latin America and the Caribbean						
Florez et al. (2003) [117]	Colombia	Middle	Mixed	115	3.5	3
Sulaiman et al. (2003) [142]	Peru	Middle	Mixed	2672	3.9	4
Chacin-Bonilla et al. (2006) [119]	Venezuela	High	Mixed	103	13.6	1
Middle East and North Africa						
Agholi et al. (2013) [127]	Iran	Middle	Mixed	356	2.2	3
Eastern Europe and central Asia						
Kucerova et al. (2011) [130]	Russia	Middle	na	46	13.0	2

*Abbreviations*: Yes, patients with diarrhea; Mixed, including patients with or without diarrhea; na, not applicable (parameter not provided)

**Table 3:** Included studies of *Isospora* infection in people with HIV listed in order of year published

	Country	Income level	Patients with diarrhea	No of patients	Prevalence (%)	Quality score
Western and central Europe and North America						
René et al. (1989) [37]	France	High	Mixed	132	0.8	2
Sorvillo et al. (1995) [25]	USA	High	Mixed	16,351	0.8	2
Mathewson et al. (1998) [42]	USA	High	Yes	83	3.6	2
Brandonisio et al. (1999) [38]	Italy	High	Mixed	154	0.6	3
Guiguet et al. (2007) [143]	France	High	Mixed	74,174	0.2	2
Lagrange-Xelot et al. (2008) [27]	France	High	Mixed	6827	0.4	1
Sub-Saharan Africa						
Henry et al. (1986) [32]	DR Congo	Low	Yes	46	19.6	0
Colebunders et al. (1988) [56]	DR Congo	Low	Yes	42	11.9	0
Therizol-Ferly et al. (1989) [57]	Ivory Coast	Middle	Yes	148	16.2	1
Hunter et al. (1992) [58]	Zambia	Middle	Mixed	90	7.8	2
Dieng et al. (1994) [60]	Senegal	Low	Yes	72	15.3	1
Fisseha et al. (1999) [63]	Ethiopia	Low	Mixed	190	1.6	2
Lebbad et al. (2001) [65]	Guinea-Bissau	Low	Yes	37	10.8	2
Keshinro et al. (2003) [26]	Nigeria	Middle	Yes	40	7.5	1
Sarfati et al. (2006) [69]	Cameroon	Middle	Mixed	154	1.9	3
Mariam et al. (2008) [70]	Ethiopia	Low	Mixed	109	1.8	2
Babatunde et al. (2010) [73]	Nigeria	Middle	Mixed	90	11.1	1
Alemu et al. (2011) [74]	Ethiopia	Low	Mixed	188	15.4	1
Wumba et al. (2012) [144]	DR Congo	Low	Mixed	242	2.9	4
Abaver et al. (2012) [145]	Nigeria	Middle	Mixed	480	1.7	3
Nwuba et al. (2012) [33]	Nigeria	Middle	Mixed	202	24.3	3
Vouking et al. (2014) [79]	Cameroon	Middle	Mixed	207	5.8	3
Girma et al. (2014) [77]	Ethiopia	Low	Mixed	268	1.5	3
Bissong et al. (2015) [80]	Cameroon	Middle	Mixed	200	6.5	3
Kiros et al. (2015) [81]	Ethiopia	Low	Mixed	399	1.3	3
Nsagha et al. (2016) [39]	Cameroon	Middle	Mixed	300	4.3	4
Shimelis et al. (2016) [3]	Ethiopia	Low	Mixed	491	2.2	3
Asia and the Pacific						
Punpoowong et al. (1998) [86]	Thailand	Middle	Yes	22	4.5	0
Wanachiwanawin et al. (1999) [87]	Thailand	Middle	Yes	91	7.7	2
Mukhopadhya et al. (1999) [146]	India	Middle	Mixed	111	12.6	1
Prasad et al. (2000) [88]	India	Middle	Yes	26	26.9	2
Waywa et al. (2001) [91]	Thailand	Middle	Yes	288	4.5	3
Wiwanitkit et al. (2001) [89]	Thailand	Middle	Mixed	60	5.0	1
Mohandas et al. (2002) [93]	India	Middle	Mixed	120	2.5	3
Kumar et al. (2002) [92]	India	Middle	Mixed	150	9.3	2
Guk et al. (2005) [95]	South Korea	High	Mixed	67	7.5	1
Dwivedi et al. (2007) [48]	India	Middle	Yes	75	2.7	2
Jayalakshmi et al. (2008) [43]	India	Middle	Yes	89	3.4	2
Saksirisampant et al. (2009) [101]	Thailand	Middle	Mixed	90	1.1	1
Kulkarni et al. (2009) [44]	India	Middle	Yes	137	8.0	1
Sherchan et al. (2012) [103]	Nepal	Low	Mixed	146	2.1	3
Baragundi Mahesh et al. (2013) [106]	India	Middle	Mixed	75	9.3	2
Vyas et al. (2013) [105]	India	Middle	Yes	75	12.0	2
Mehta et al. (2013) [104]	India	Middle	Mixed	100	18.0	3
Gupta et al. (2013) [45]	India	Middle	Mixed	100	25.0	2
Jain et al. (2014) [108]	India	Middle	Mixed	250	0.8	2
Paboriboune et al. (2014) [107]	Laos	Middle	Mixed	137	4.4	3
Khalil et al. (2015) [110]	India	Middle	Mixed	200	7.5	3
Kaniyarakkal et al. (2016) [112]	India	Middle	Mixed	200	4.5	2
Mitra et al. (2016) [113]	India	Middle	Mixed	194	14.4	2
Shah et al. (2016) [114]	India	Middle	Mixed	45	20.0	2
Latin America and the Caribbean						
Escobedo et al. (1999) [116]	Cuba	Middle	Mixed	67	1.5	2
Moran et al. (2005) [147]	Mexico	Middle	Mixed	203	0.5	3
Cardoso et al. (2011) [121]	Brazil	Middle	Mixed	500	1.2	3
Guimarães et al. (2012) [123]	Brazil	Middle	Mixed	93	1.1	1
Assis et al. (2013) [124]	Brazil	Middle	Mixed	59	6.8	2
Middle East and North Africa						
Agholi et al. (2013) [127]	Iran	Middle	Mixed	356	0.6	3
Salehi Sangani et al. (2016) [128]	Iran	Middle	Mixed	80	2.5	2

*Abbreviations*: Mixed, including patients with or without diarrhea; Yes, patients with diarrhea

**Fig. 2:**
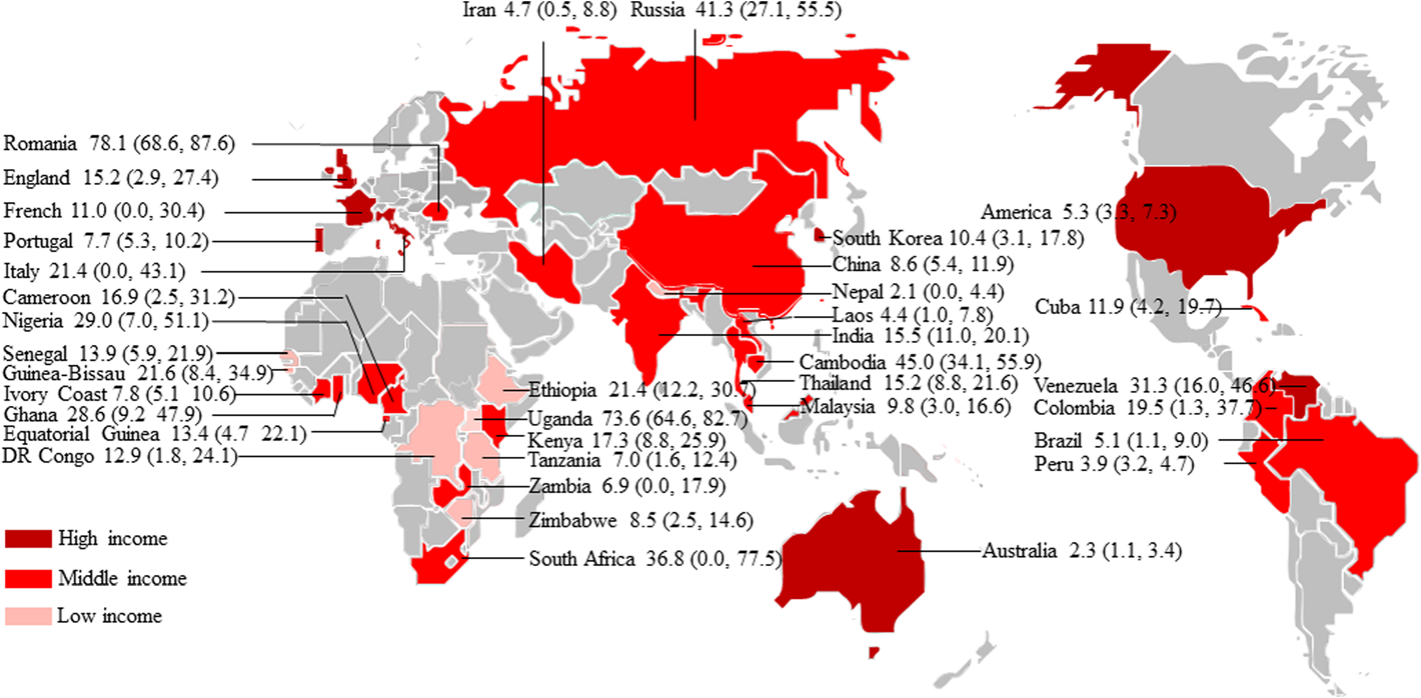
Map of *Cryptosporidium* infection in HIV-infected people worldwide. Pooled percentage prevalence and 95% CI are shown for each country

One hundred and six studies assessed *Cryptosporidium* infection in HIV-infected people (Fig. 1, Table 1), including a total of 43,218 HIV-infected patients. These studies were done in 36 countries (Fig. 2), including five countries of western and central Europe and North America, 15 of sub-Saharan Africa, four of Latin America and the Caribbean, two of eastern Europe and central Asia, nine of Asia and the Pacific, and one of Middle East and North Africa. Of these identified studies, 16 were done in low-income countries, 76 were in middle-income countries, and 14 were in high-income countries (Fig. 2). Ninety-eight papers were written in English, and eight in Chinese [16–23].

**Fig. 3:**
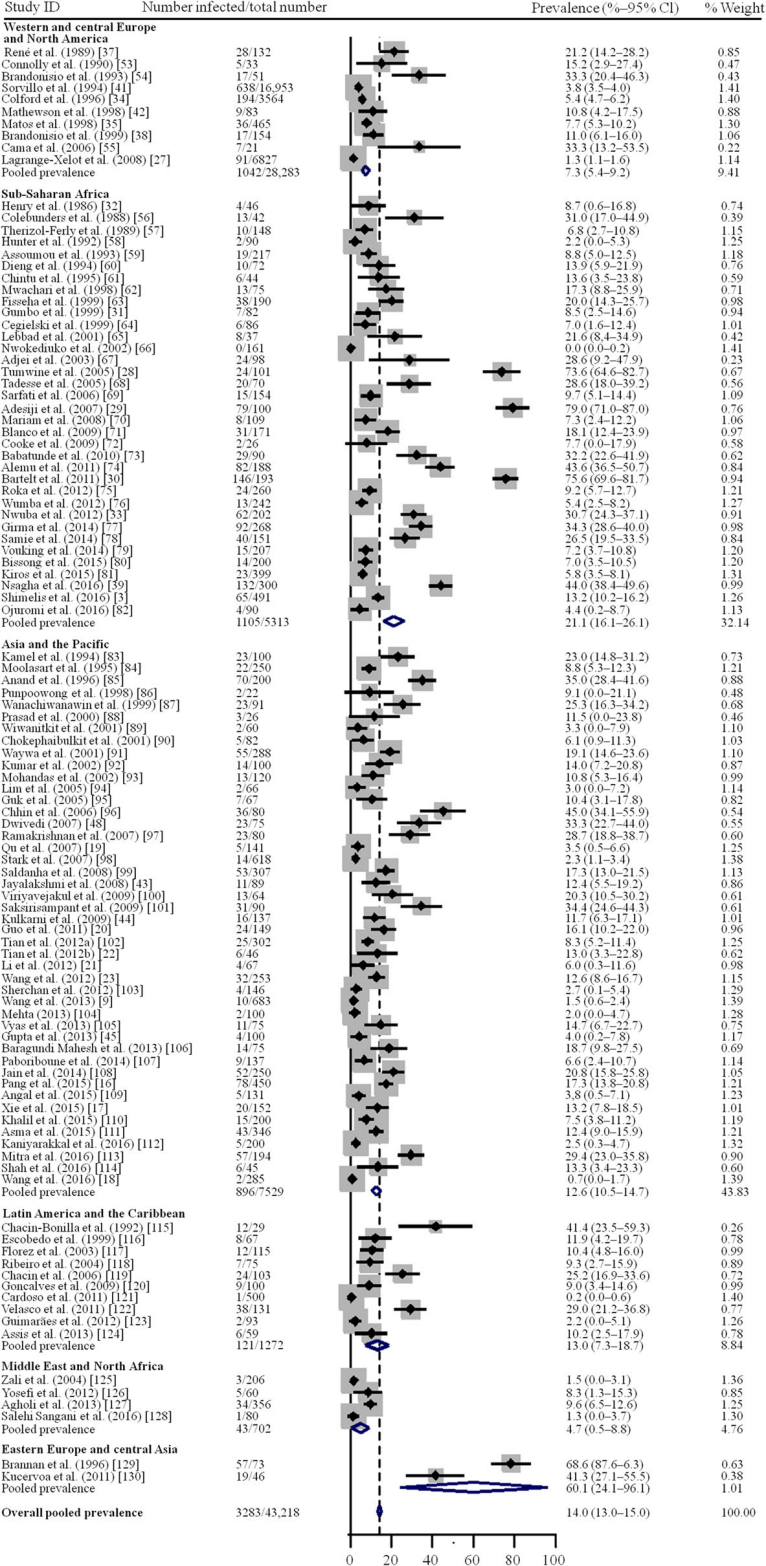
Random-effect meta-analysis of *Cryptosporidium* infection in HIV-infected people

The prevalence of *Cryptosporidium* infection ranged between 0 and 78.1% (Fig. 3). Meta-analysis by random-effect model showed that the estimated pooled prevalence of *Cryptosporidium* infection in people with HIV infection was 14.0% (3283/43,218; 95% CI: 13.0–15.0%) overall, 21.1% (1105/5315; 95% CI: 16.1–21.1%) in sub-Saharan Africa, 7.3% (1042/28,283; 95% CI: 5.4–9.2%) in western and central Europe and North America, 12.6% (896/7529; 95% CI: 10.5–14.7%) in Asia and the Pacific, 13.0% (121/1272; 95% CI: 7.3–18.7%) in Latin America and the Caribbean, 4.7% (43/702; 95% CI: 0.5–8.8%) in the Middle East and North Africa, and 60.1% (76/119; 95% CI: 24.1–96.1%) in eastern Europe and central Asia. Only four studies were done in Middle East and North Africa, and two in eastern Europe and central Asia, where the prevalence of *Cryptosporidium* infection in HIV-infected people was very poorly recorded.

With a substantial heterogeneity (*I*^2^ = 97.6%, *P* < 0.0001; Table 4), meta-regression analyses showed that geographical distribution (*P* = 0.039) and patients with diarrhea (*P* = 0.009) might be sources of heterogeneity, whereas we detected no significant differences in income levels (*P* = 0.328). Subgroup analysis showed the pooled prevalence of *Cryptosporidium* infection in HIV-infected people was significantly lower in western and central Europe and North America than in sub-Saharan Africa (OR 0.73, 95% CI: 0.54–0.99, *P* = 0.044), and higher in patients with diarrhea (OR 1.21, 95% CI: 1.00–1.46, *P* = 0.047).

**Table 4:** Pooled prevalence of *Cryptosporidium* infection in HIV-infected patients

	No. of studies	No. of HIV-infected patients	No. of patients with *Cryptosporidium* co-infection	Prevalence of *Cryptosporidium* co-infection (95% CI) (%)	Heterogeneity		Univariate meta-regression	
					*P-*value	*I*^2^ (%)	Coefficient (95% CI) (%)	*P-*value
Region							0.20 (0.01–0.38)	0.039
Western and central Europe and North America	10	28,283	1042	7.3 (5.4–9.2)	< 0.0001	97.0		
Sub-Saharan Africa	35	5313	1105	21.1 (16.1–26.1)	< 0.0001	98.5		
Asia and the Pacific	45	7529	896	12.6 (10.5–14.7)	< 0.0001	94.1		
Latin America and the Caribbean	10	1272	121	13.0 (7.3–18.7)	< 0.0001	94.0		
Middle East and North Africa	4	702	43	4.7 (0.5–8.8)	< 0.0001	88.1		
Eastern Europe and central Asia	2	119	76	60.1 (24.1–96.1)	< 0.0001	94.4		
Income level					< 0.0001		0.12 (-0.12–0.37)	0.328
Low income	16	2559	460	19.7 (13.3–26.1)	< 0.0001	96.6		
Middle income	76	11,559	1722	14.8 (13.3–16.4)	< 0.0001	97.5		
High income	14	29,100	1101	7.7 (6.0–9.5)	< 0.0001	96.2		
Patients with diarrhea					< 0.0001		0.19 (0.05–0.33)	0.009
Yes	34	4232	625	18.2 (14.6–21.7)	< 0.0001	97.3		
Mixed	66	37,517	2306	11.8 (10.6–13.0)	< 0.0001	96.7		
na	6	1469	352	29.4 (12.4–46.4)	< 0.0001	98.7		
Total	106	43,218	3283	14.0 (13.0–15.0)	< 0.0001	97.6		

*Abbreviations*: Yes, patients with diarrhea; Mixed, including patients with or without diarrhea; na, not applicable (parameter not provided)

**Fig. 4:**
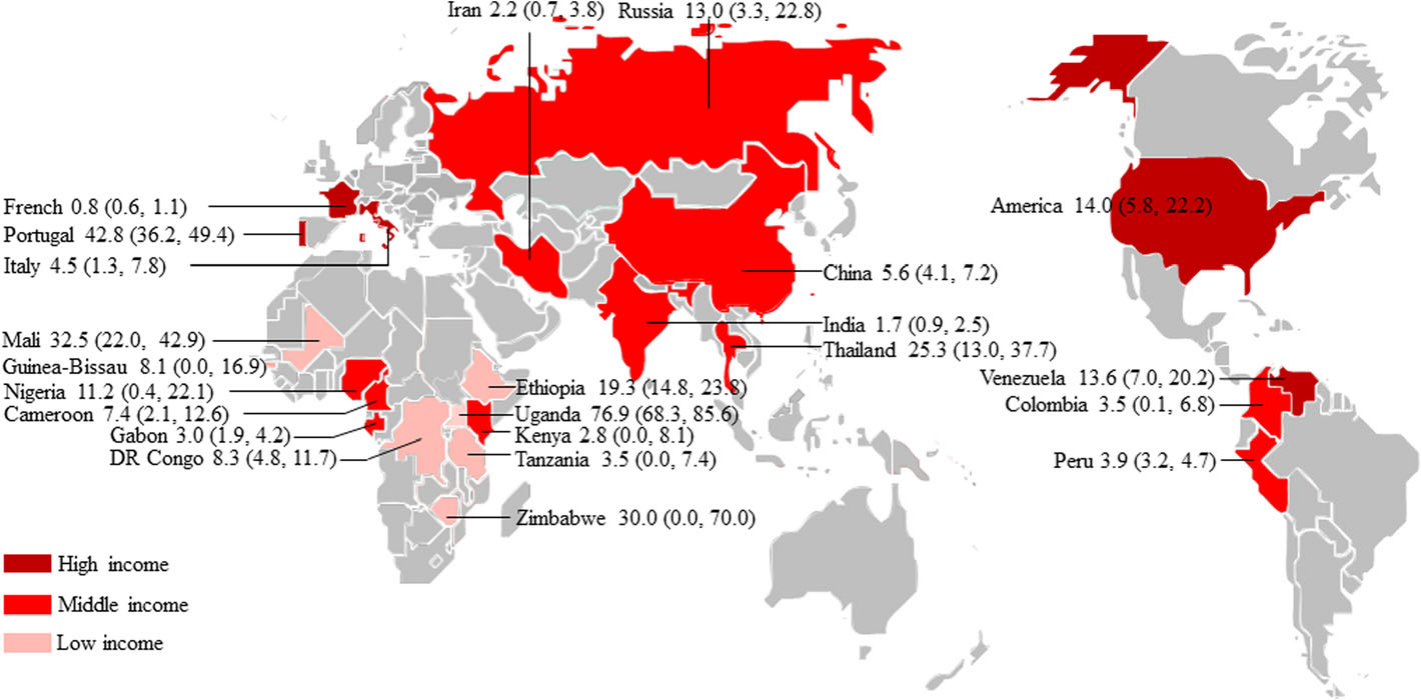
Map of microsporidia infection in HIV-infected people worldwide. Pooled percentage prevalence and 95% CI are shown for each country

Forty-seven studies reported prevalence of microsporidia (Fig. 1, Table 2), including a total of 18,006 HIV-infected people tested for microsporidia infection. The included studies were conducted in 23 countries (Fig. 4), including 11 countries of sub-Saharan Africa, four of western and central Europe and North America, three of Asia and the Pacific, three of Latin America and the Caribbean, one each of Middle East and North Africa and eastern Europe and central Asia. Of the identified studies, 9 were done in low-income countries, 30 were in middle-income countries, and 9 were in high-income countries (Fig. 4). Forty-five papers were written in English, one each in Chinese and French [17, 24].

The prevalence of microsporidia infection ranged between 0.7–81.3% (Additional file 2: Figure S1). Meta-analysis by random-effect model indicated that the estimated pooled prevalence of microsporidia infection in people with HIV infection was 11.8% (1090/18,006; 95% CI: 10.1–13.4%) overall, 15.4% (425/3834; 95% CI: 11.1–19.7%) in sub-Saharan Africa, 14.4% (277/8089; 95% CI: 7.8–21.1%) in western and central Europe and North America, 11.7% (251/2791; 95% CI: 8.2–15.1%) in Asia and the Pacific, 5.6% (123/2890; 95% CI: 1.9–9.3%) in Latin America and the Caribbean, 2.2% (8/356; 95% CI: 0.7–3.8%) in the Middle East and North Africa, and 13.0% (6/46; 95% CI: 3.3–22.8%) in eastern Europe and central Asia. Only three studies were done in Latin America and the Caribbean, one each in Middle East and North Africa, and in eastern Europe and central Asia. The prevalence of microsporidia infection in these regions should be interpreted with caution.

Due to the substantial heterogeneity (*I*^2^ = 96.7%, *P* < 0.0001; Table 5), meta-regression analyses indicated that the income level (*P* = 0.024) and patients with diarrhea (*P* = 0.004) might be sources of heterogeneity, whereas we detected no significant differences in geographical distribution (*P* = 0.323). Subgroup analysis showed the pooled prevalence of microsporidia infection in HIV-infected people was significantly higher in low-income countries than in middle-income countries (OR 1.58, 95% CI: 1.08–2.31, *P* = 0.018), and higher in patients with diarrhea than the control (OR 1.54, 95% CI: 1.14–2.07, *P* = 0.005).

**Table 5:** Pooled prevalence of microsporidia infection in HIV-infected patients

	No. of studies	No. of HIV-infected patients	No. of patients with microsporidia co-infection	Prevalence of microsporidia co-infection (95% CI) (%)	Heterogeneity		Univariate meta-regression	
					*P-*value	*I*^2^ (%)	Coefficient (95% CI) (%)	*P-*value
Region							0.16 (0.16–0.47)	0.323
Western and central Europe and North America	8	8089	277	14.4 (7.8–21.1)	< 0.0001	97.6		
Sub-Saharan Africa	17	3834	425	15.4 (11.1–19.7)	< 0.0001	96.9		
Asia and the Pacific	18	2791	251	11.7 (8.2–15.1)	< 0.0001	95.8		
Latin America and the Caribbean	3	2890	123	5.6 (1.9–9.3)	0.017	75.6		
Middle East and North Africa	1	356	8	2.2 (0.7–3.8)	–	–		
Eastern Europe and central Asia	1	46	6	13.0 (3.3–22.8)	–	–		
Income level							0.42 (0.06–0.79)	0.024
Low income	9	1011	219	25.2 (13.0–37.4)	< 0.0001	97.3		
Middle income	30	8803	580	8.4 (6.5–10.3)	<0.0001	94.5		
High income	9	8192	291	14.4 (8.1–20.6)	< 0.0001	97.4		
Patients with diarrhea							0.44 (0.15–0.73)	0.004
Yes	17	1807	396	22.2 (14.5–29.9)	< 0.0001	96.9		
Mixed	28	14,573	641	8.3 (6.5–10.1)	< 0.0001	96.3		
na	3	1626	53	3.2 (1.7–4.6)	0.128	51.3		
Total	48	18,006	1090	11.8 (10.1–13.4)	< 0.0001	96.7		

*Abbreviations*: Yes, patients with diarrhea; Mixed, including patients with or without diarrhea; na, not applicable (parameter not provided)

**Fig. 5:**
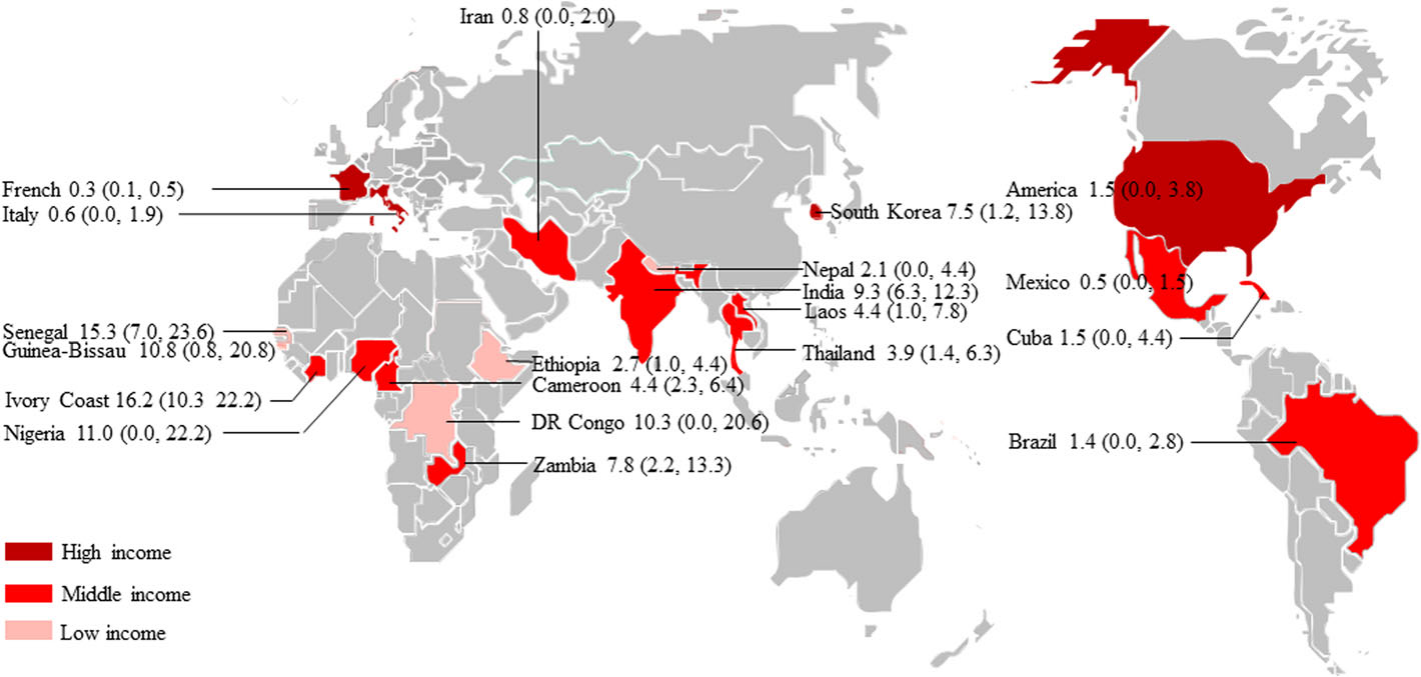
Map of *Isospora* infection in HIV-infected people worldwide. Pooled percentage prevalence and 95% CI are shown for each country

Fifty-eight studies tested 105,922 HIV-infected patients for *Isospora* infection (Fig. 1, Table 3). The selected studies were done in 20 countries (Fig. 5), including three countries of western and central Europe and North America, eight of sub-Saharan Africa, five of Asia and the Pacific, three of Latin America and the Caribbean, and one of Middle East and North Africa. No studies were found from eastern Europe and central Asia. Of the identified studies, 12 were done in low-income countries, 39 were in middle-income countries, and seven were in high-income countries (Fig. 5). All the included papers were written in English.

The prevalence of *Isospora* infection ranged between 0.2–26.9% (Additional file 3: Figure S2). Meta-analysis by random-effect model showed that the estimated pooled prevalence of *Isospora* infection in people with HIV infection was 2.5% (788/105,922; 95% CI: 2.1–2.9%) overall, 6.1% (232/3995; 95% CI: 4.5–7.7%) in sub-Saharan Africa, 0.5% (324/97,721; 95% CI: 0.2–0.8%) in western and central Europe and North America, 7.1% (215/2848; 95% CI: 5.2–9.0%) in Asia and the Pacific, 1.0% (13/922; 95% CI: 0.3–1.7%) in Latin America and the Caribbean, 0.8% (4/436; 95% CI: 0–2.0%) in the Middle East and North Africa. However, few data were available from Latin America, Middle East and North Africa. Only two studies were conducted in Middle East and North Africa, five were done in Latin America and the Caribbean, showing a poor record of *Isospora* infection in these regions.

With a substantial heterogeneity (*I*^2^ = 89.8%, *P* < 0.0001; Table 6), meta-regression analyses showed that patients with diarrhea might be sources of heterogeneity (*P* = 0.005), whereas we detected no significant differences in region distribution (*P* = 0.143) and income levels (*P* = 0.806). Subgroup analysis showed that the pooled prevalence of *Isospora* infection in HIV-infected people was significantly lower in central Europe and North America than in sub-Saharan Africa (OR 0.40, 95% CI: 0.27–0.59) and in Asia and the Pacific (OR 0.37, 95% CI: 0.26–0.54). Additionally, it was significantly higher in low-income countries (OR 1.94, 95% CI: 1.24–3.04, *P* = 0.005) and middle-income countries (OR 2.08, 95% CI: 1.41–3.07, *P* < 0.0001) than in high-income countries. We also found that patients with diarrhea had a higher prevalence of *Isospora* infection (OR 1.53, 95% CI: 1.14–2.06, *P* = 0.005).

**Table 6:** Pooled prevalence of *Isospora* infection in HIV-infected patients

	No. of studies	No. of HIV-infected patients	No. of patients with *Isospora* co-infection	Prevalence of *Isospora* co-infection (95% CI) (%)	Heterogeneity		Univariate meta-regression	
					*P-*value	*I*^2^ (%)	Coefficient (95% CI) (%)	*P-*value
Region							0.21 (-0.07–0.49)	0.143
Western and central Europe and North America	6	97,721	324	0.5 (0.2–0.8)	< 0.0001	92.8		
Sub-Saharan Africa	21	3995	232	6.1 (4.5–7.7)	< 0.0001	87.0		
Asia and the Pacific	24	2848	215	7.1 (5.2–9.0)	< 0.0001	83.4		
Latin America and the Caribbean	5	922	13	1.0 (0.3–1.7)	0.349	10.1		
Middle East and North Africa	2	436	4	0.8 (0.0–2.0)	0.279	14.7		
Income level							-0.04 (-0.38–0.30)	0.806
Low income	12	2230	93	3.8 (2.2–5.5)	< 0.0001	91.9		
Middle income	39	5904	366	5.8 (4.7–7.0)	< 0.0001	86.9		
High income	7	97,788	329	0.5 (0.2–0.9)	< 0.0001	80.0		
Patients with diarrhea					< 0.0001		-0.43 (-0.72– -0.13)	0.005
Yes	15	1271	112	8.3 (5.7–10.9)	< 0.0001	66.1		
Mixed	43	104,651	676	2.0 (1.6–2.4)	< 0.0001	90.4		
Total	58	105,922	788	2.5 (2.1–2.9)	< 0.0001	89.8		

*Abbreviations*: Yes, patients with diarrhea; Mixed, including patients with or without diarrhea

We determined the effect of selected studies on the pooled prevalence by excluding single studies sequentially, and found no significant effect of study quality on prevalence of *Cryptosporidium* and microsporidia infection in HIV-infected people (all *P* > 0.05), but there was significant effect of study quality on the prevalence of *Isospora* infection (*P* = 0.033 and 0.043).

When we excluded the studies by Sorvillo et al. [25], Guiguet et al. [26], and Lagrange-Xelot et al. [27], the pooled prevalence of *Isospora* infection in HIV-infected people was increased from 2.5% (95% CI: 2.1–2.9%) to 3.0% (95% CI: 2.5–3.5%), 3.3% (95% CI: 2.8–3.8%), and 3.0% (95% CI: 2.5–3.4%), respectively. These findings indicated that the pooled prevalence of *Isospora* infection in HIV-infected people was substantially influenced by the three studies, and adjusted to 5.0% (469/8570; 95% CI: 4.1–5.9%) by excluding these studies (Additional file 4: Figure S3).

## Discussion

Our aim was to estimate the worldwide prevalence of opportunistic intestinal protozoa in people with HIV, showing that *Cryptosporidium* and microsporidia are the main intestinal protozoa in HIV-infected people, followed by *Isospora*; their prevalences are usually high in sub-Saharan Africa and in patients with diarrhea, and low in high-income countries. Because of the large proportion of low-income countries and the large number of people with HIV [12], sub-Saharan Africa has a very high burden of *Cryptosporidium*, microsporidia and *Isospora* infection, reinforcing the importance of routine testing for opportunistic intestinal protozoa in all HIV-infected people. To our knowledge, this is the first systematic review and meta-analysis of the global prevalence of *Cryptosporidium*, microsporidia and *Isospora* infection in HIV-infected people.

Our findings corroborate evidence for a high prevalence of *Cryptosporidium*, microsporidia and *Isospora* infection in Africa and a low prevalence in Europe. In HIV-infected people, a high prevalence has been reported in Uganda (73.6%) [28], Nigeria (79.0%) [29], and South Africa (75.6%) [30] for *Cryptosporidium* infection; in Zimbabwe (50.9%) [31] and Uganda (76.9%) [28] for microsporidia infection; and in DR Congo (19.6%) [32] and Nigeria (24.3%) [33] for *Isospora* infection. In contrast, a low prevalence has been shown in France (1.3%) [27], USA (5.4%) [34] and Portugal (7.7%) [35] for *Cryptosporidium* infection; in France (0.8%) [27] and USA (5.9%) [36] for microsporidia infection; and in France (0.8%) [37] and Italy (0.6%) [38] for *Isospora* infection.

The incidence of opportunistic intestinal protozoa infection varies, relying on sanitation facilities, drinking contaminated water, animal exposure, CD4 T cell count, ART, diagnostic methods [39, 40]. Thus, the prevalence of infection may vary substantially, even within a country or among different populations of the same region. For example, in the USA, the prevalence of *Cryptosporidium* infection is 3.8% in Los Angeles [41], 5.4% in San Francisco [34] and 10.8% in Houston [42]. Large differences of *Isospora* infection have also been reported in India, with a prevalence of 3.4% in Coimbatore [43], 8.0% in Pune [44] and 25.0% in New Delhi [45]. There are significant differences between different countries for *Cryptosporidium* (0–78.1%), microsporidia (0.7–81.3%) and *Isospora* (0.2–26.9%) infection in HIV-infected people. However, limited country-level surveys of *Cryptosporidium*, microsporidia and *Isospora* infection have been undertaken, making it difficult to compare the infections between regions or populations.

The majority of the studies had additional data on opportunistic intestinal protozoa. Due to the variability of data quality and reporting consistency, we only extracted and analyzed the data on diarrhea, and demonstrated it was related to *Cryptosporidium* (OR: 1.21, 95% CI: 1.01–1.46, *P* = 0.047), microsporidia (OR 1.53, 95% CI: 1.13–2.07, *P* = 0.007) and *Isospora* (OR 1.53, 95% CI: 1.14–2.06, *P* = 0.005) infection in HIV-infected people in comparison with their controls. Moreover, there were some case-control studies that investigated opportunistic intestinal protozoa infection in people with HIV with and without diarrhea. We analyzed the association of diarrhea with *Cryptosporidium*, microsporidia and *Isospora* infection in HIV-infected people. The estimated pooled random effects ORs of *Cryptosporidium*, microsporidia and *Isospora* infection in HIV people with diarrhea compared with their controls were 4.09 (95% CI: 2.32–7.20), 4.72 (95% CI: 3.47–6.42), and 4.93 (95% CI: 3.33–7.29), respectively (Additional files 5, 6 and 7: Figures S4, S5 and S6). These findings show that diarrhea is associated with opportunistic intestinal protozoa infection in HIV people. However, other factors seem to increase the likelihood of infection with opportunistic intestinal protozoa, including CD4 T-lymphocyte counts of less than 100 cells/μl [46], ingestion of contaminated drinking water or food [47], exposure to infected pets or animals [48] and unsafe homosexual activity [49].

There are a few limitations of the present meta-analysis, which may affect the results. First, many relevant studies were identified through our literature search, but not all data were available; there is a possibility that some qualified data were missed. Secondly, the majority of the studies were of moderate or low quality, as most of the data resulted from the conventional microscopic diagnostic techniques; these have a sensitivity which is inferior to polymerase chain reaction, ELISA and direct fluorescent-antibody tests. Additionally, most studies examined a single stool specimen, potentially leading to a false negative result. This means that the reported prevalence was possibly underestimated. Thirdly, the included studies were concentrated in Asia (*n* = 50), sub-Saharan Africa (*n* = 45), and western and central Europe and North America (*n* = 17), Latin America and the Caribbean (*n* = 12), with few studies from Middle East and North Africa (*n* = 5), and eastern Europe and central Asia (*n* = 2), and the study quality was variable, emphasizing the need for more robust surveillance of *Cryptosporidium*, microsporidia and *Isospora* infection in HIV-infected people in these regions. Fourthly, different species and genotypes o*f Cryptosporidium* and microsporidia may cause different clinical manifestations in HIV-infected people [40, 50]. However, we did not analyze their distribution characteristics as the microscopic diagnostic techniques in most of the selected studies could not identify the species within the genus *Cryptosporidium* and microsporidians.

To explain the specific causes of heterogeneity, we did univariate meta-regression analyses on various sources including geographical distribution, income level, and patients with diarrhea, and found different main causes of heterogeneity for the three opportunistic protozoa. These may come from geographical distribution (*P* = 0.039) and patients with diarrhea (*P* = 0.009) for *Crytosporidium* infection, from income level (*P* = 0.024) and patients with diarrhea (*P* = 0.004) for microsporidia infection, and from patients with diarrhea (*P* = 0.005) for *Isospor*a infection. Other potential causes of heterogeneity may include publication year, sample size, and detection methods. Unfortunately, we did not analyze them, as there were not enough data available.

Moreover, we did dummy variable analysis on geographical distribution, income level, and patients with diarrhea. The countries in sub-Saharan Africa had a higher prevalence of *Cryptosporidium* and *Isospora* infection in HIV-infected patients than those in western and central Europe and North America, and the low-income countries had a higher prevalence of microsporidia and *Isospora* infection than the middle or high-income countries. These findings support an association between parasite infection and the income level of countries, which could be due to the fact that people in high-income countries have access to safe water and sanitation facilities, which are responsible for the reduced odds of parasite infection.

## Conclusions

The results of our global meta-analysis show a heavy burden of *Cryptosporidium*, microsporidia and *Isospora* infection in HIV-infected people, especially in low-income countries and sub-Saharan Africa. Thus, routine screening of opportunistic intestinal protozoa should be done, particularly for those who have CD4 T-lymphocyte count less than 100 cells/μl, and early treatment should be administered. This should include a combination of antibiotics of azithromycin, paramomycin and nitazoxanide for *Cryptosporidium* infection, albendazole for microsporidia infection, and trimethoprim-sulfamethoxazole for *Isospora* infection [51, 52]. However, antibiotics alone may not necessarily reduce the symptoms associated with opportunistic intestinal protozoa infection [7, 51]. More importantly, it is obligatory to reconstruct the immune system by ART. Additional preventive measures should also emphasize the environmental and personal hygiene, along with the quality of drinking water [47].

## Additional files

Additional file 1: Table S1. Checklist of items to include when reporting a meta-analysis. (DOC 69 kb)

Additional file 2: Figure S1. Random-effect meta-analysis of microsporidia infection in HIV-infected people. (PDF 267 kb)

Additional file 3: Figure S2. Random-effect meta-analysis of *Isospora* infection in HIV-infected people. (PDF 345 kb)

Additional file 4: Figure S3. Random-effect meta-analysis of *Isospora* infection in HIV-infected people when the three studies affecting the prevalence of *Isospora* were excluded. (PDF 344 kb)

Additional file 5: Figure S4. Random-effect meta-analysis of the association of diarrhea with *Cryptosporidium* infection in HIV-infected people. (PDF 227 kb)

Additional file 6: Figure S5. Fixed-effect meta-analysis of the association of diarrhea with microsporidia infection in HIV-infected people. (PDF 170 kb)

Additional file 7: Figure S6. Fixed-effect meta-analysis of the association of diarrhea with *Isospora* infection in HIV-infected people. (PDF 217 kb)

## Abbreviations

AIDS: Acquired immune deficiency syndrome
ART: Antiretroviral therapy
CI: Confidence interval
HIV: Human immunodeficiency virus
OR: Odds ratio
PRISMA: Preferred reporting items for systematic reviews and meta-analyses
