# Supplementary material for: Prevalence of Cryptosporidium, microsporidia and Isospora infection in HIV-infected people: a global systematic review and meta-analysis
Source: Parasit Vectors. 2018 Jan 9;11:28. doi: 10.1186/s13071-017-2558-x (PMC5759777; doi:10.1186/s13071-017-2558-x)

Additional file 2: Figure S1. Random-effect meta-analysis of microsporidian infection in HIV-infected people.

Study ID	Number infected/ total number	Prevalence (%–95% CI)	% Weight
**Western and central Europe and North America**			
Weber et al. (1992) [131]	6/134	4.5 (1.0–8.0)	2.37
Kotler et al. (1994) [132]	56/141	28.9 (22.5–35.2)	1.91
Anwar-Bruni et al. (1996) [36]	22/371	5.9 (3.5–8.3)	2.50
Coyle et al. (1996) [133]	31/111	27.9 (19.6–36.3)	1.60
Mathewson et al. (1998) [42]	5/83	6.0 (0.9–11.1)	2.12
Brandonisio et al. (1999) [38]	7/154	4.5 (1.3–7.8)	2.40
Ferreira et al. (2001) [134]	92/215	42.8 (36.2–49.4)	1.87
Lagrange-Xelot et al. (2008) [27]	58/6827	0.8 (0.6–1.1)	2.64
Pooled prevalence	277/8089	14.4 (7.8–21.1)	17.41
**Sub-Saharan Africa**			
van Gool et al. (1995) [135]	13/129	10.1 (4.9–15.3)	2.11
Maiga et al. (1997) [24]	25/77	32.5 (22.0–42.9)	1.31
Mwachari et al. (1998) [62]	1/36	2.8 (0.0–8.1)	2.08
Cegielski et al. (1999) [64]	3/86	3.5 (0.0–7.4)	2.31
Gumbo et al. (1999) [31]	28/55	50.9 (37.7–64.1)	1.01
Lebbad et al. (2001) [65]	3/37	8.1 (0.0–16.9)	1.53
Endeshaw et al. (2005) [136]	18/80	22.5 (13.3–31.7)	1.48
Tumwine et al. (2005) [28]	70/91	76.9 (68.3–85.6)	1.55
Endeshaw et al. (2006) [137]	39/214	18.2 (13.1–23.4)	2.11
Sarfati et al. (2006) [69]	8/154	5.2 (1.7–8.7)	2.37
Breton et al. (2007) [138]	25/822	3.0 (1.9–4.2)	2.60
Breton et al. (2007) [138]	22/758	2.9 (1.7–4.1)	2.60
Akinbo et al. (2012) [139]	77/463	16.6 (13.2–20.0)	2.38
Wumba et al. (2012) [76]	20/242	8.3 (4.8–11.7)	2.37
Bissong et al. (2015) [80]	4/200	2.0 (0.1–3.9)	2.55
Nsagha et al. (2016) [39]	64/300	21.3 (16.7–26.0)	2.20
Ojuromi et al. (2016) [82]	4/90	5.6 (0.8–10.3)	2.18
Pooled prevalence	425/3834	15.4 (11.1–19.7)	34.75
**Asia and the Pacific**			
Punpoowong et al. (1998) [86]	6/22	27.3 (8.7–45.9)	0.63
Wanachiwanawin et al. (1998) [140]	22/66	33.3 (22.0–44.7)	1.20
Wanachiwanawin et al. (1999) [87]	26/91	28.6 (19.3–37.9)	1.46
Chokephaibulkit et al. (2001) [90]	16/82	19.5 (10.9–28.1)	1.57
Wiwanitkit et al. (2001) [89]	1/60	1.7 (0.0–4.9)	2.40
Waywa et al. (2001) [91]	28/288	9.7 (6.3–13.1)	2.38
Kumar et al. (2002) [92]	1/150	0.7 (0.0–2.0)	2.60
Wanachiwanawin et al. (2002) [141]	24/95	25.3 (16.5–34.0)	1.54
Mohandas et al. (2002) [93]	3/120	2.5 (0.0–5.3)	2.46
Dwivedi et al. (2007) [48]	5/75	6.7 (1.0–12.3)	2.03
Saksirisampant et al. (2009) [101]	5/90	5.6 (0.8–10.3)	2.18
Viriyavejakul et al. (2009) [100]	52/64	81.3 (71.7–90.8)	1.43
Kulkarni et al. (2009) [44]	2/137	1.5 (0.0–3.5)	2.54
Wang et al. (2013) [9]	39/683	5.7 (4.0–7.5)	2.56
Xie et al. (2015) [17]	8/152	5.3 (1.7–8.8)	2.36
Khalil et al. (2015) [110]	5/200	2.5 (0.3–4.7)	2.53
Khanduja et al. (2016) [8]	4/222	1.8 (0.1–3.6)	2.56
Mitra et al. (2016) [113]	4/194	2.1 (0.1–4.1)	2.54
Pooled prevalence	251/2791	11.7 (8.2–15.1)	36.97
**Latin America and the Caribbean**			
Florez et al. (2003) [117]	4/115	3.5 (0.1–6.8)	2.39
Sulaiman et al. (2003) [142]	105/2672	3.9 (3.2–4.7)	2.62
Chacin-Bonilla et al. (2006) [119]	14/103	13.6 (7.0–20.2)	1.87
Pooled prevalence	123/2890	5.6 (1.9–9.3)	6.89
**Middle East and North Africa**			
Agholi et al. (2013) [127]	8/356	2.2(0.7–3.8)	2.58
Pooled prevalence	8/356	2.2 (0.7–3.8)	2.58
**Eastern Europe and central Asia**			
Kucerova et al. (2011) [130]	6/46	13.0 (3.3–22.8)	1.40
Pooled prevalence	6/46	13.0 (3.3–22.8)	1.40
**Overall pooled prevalence**	**1090/18,006**	**11.8 (10.1–13.4)**	**100.00**

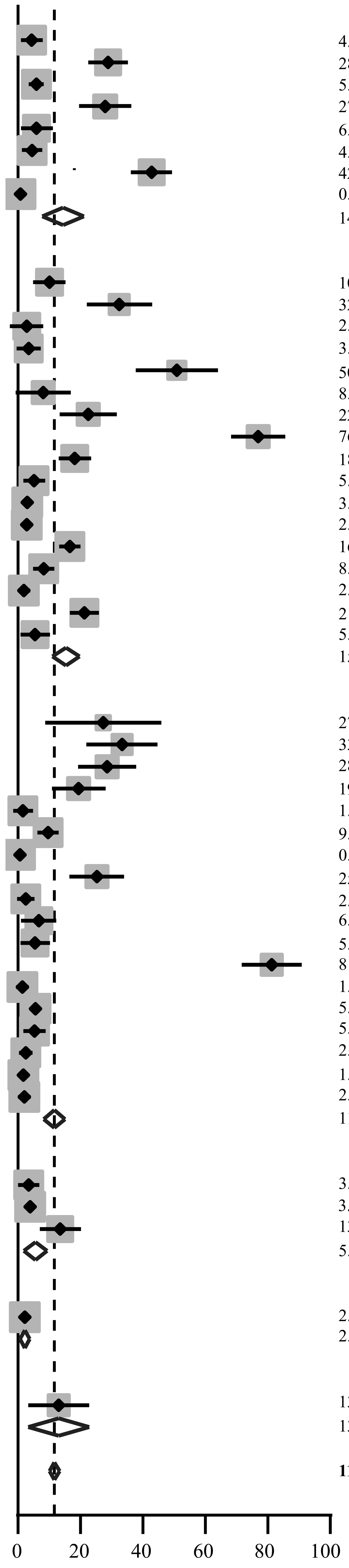

Supplement: Supplementary file 2 — Random-effect meta-analysis of microsporidia infection in HIV-infected people. (PDF 267 kb) [file 13071_2017_2558_MOESM2_ESM.pdf]
