# Supplementary material for: Prevalence of Cryptosporidium, microsporidia and Isospora infection in HIV-infected people: a global systematic review and meta-analysis
Source: Parasit Vectors. 2018 Jan 9;11:28. doi: 10.1186/s13071-017-2558-x (PMC5759777; doi:10.1186/s13071-017-2558-x)

Additional file 3: Figure S2. Random-effect meta-analysis of *Isospora* infection in HIV-infected people.

Study ID	Number infected/ total number	Prevalence (%–95% Cl)	% Weight
**Western and central Europe and North America**			
René et al. (1989) [37]	1/132	0.8 (0.0–2.2)	3.33
Sorvillo et al. (1995) [25]	127/16,351	0.8 (0.6–0.9)	6.25
Mathewson et al.(1998) [42]	3/83	3.6 (0.0–7.6)	0.83
Brandonisio et al. (1999) [38]	1/154	0.6 (0.0–1.9)	3.80
Guiguet et al. (2007) [143]	164/74,174	0.2 (0.2–0.3)	6.30
Lagrange-Xelot et al. (2008) [27]	28/6827	0.4 (0.3–0.6)	6.24
Pooled prevalence	324/97,721	0.5 (0.2–0.8)	26.75
**Sub-Saharan Africa**			
Henry et al. (1986) [32]	9/46	19.6 (8.1–31.0)	0.12
Colebunders et al. (1988) [56]	5/42	11.9 (2.1–21.7)	0.16
Therizol-Ferly et al. (1989) [57]	24/148	16.2 (10.3–22.2)	0.41
Hunter et al. (1992) [58]	7/90	7.8 (2.2–13.3)	0.47
Dieng et al. (1994) [60]	11/72	15.3 (7.0–23.6)	0.22
Fisseha et al.(1999) [63]	3/190	1.6 (0.0–3.4)	2.76
Lebbad et al.(2001) [65]	4/37	10.8 (0.8–20.8)	0.15
Keshinro et al. (2003) [26]	3/40	7.5 (0.0–15.7)	0.22
Sarfati et al. (2006) [69]	3/154	1.9 (0.0,4.1)	2.14
Mariam et al. (2008) [70]	2/109	1.8 (0.0–4.4)	1.76
Babatunde et al. (2010) [73]	10/90	11.1 (4.6–17.6)	0.35
Alemu et al. (2011) [74]	29/188	15.4 (10.3–20.6)	0.53
Wumba et al. (2012) [144]	7/242	2.9 (0.8–5.0)	2.24
Abaver et al. (2012) [145]	8/480	1.7 (0.5–2.8)	4.11
Nwuba et al. (2012) [33]	49/202	24.3 (18.3–30.2)	0.41
Vouking et al. (2014) [79]	12/207	5.8 (2.6–9.0)	1.23
Girma et al. (2014) [77]	4/268	1.5 (0.0–2.9)	3.39
Bissong et al. (2015) [80]	13/200	6.5 (3.1–9.9)	1.09
Kiros et al. (2015) [81]	5/399	1.3 (0.2–2.3)	4.24
Nsagha et al. (2016) [39]	13/300	4.3 (2.0–6.6)	1.99
Shimelis et al. (2016) [3]	11/491	2.2 (0.9–3.5)	3.71
Pooled prevalence	232/3995	6.1 (4.5–7.7)	31.69
**Asia and the Pacific**			
Punpoowong et al.(1998) [86]	1/22	4.5 (0.0–13.2)	0.20
Wanachiwanawin et al.(1999) [87]	7/91	7.7 (2.2–13.2)	0.48
Mukhopadhya et al. (1999) [146]	14/111	12.6 (6.4–18.8)	0.38
Prasad et al. (2000) [88]	7/26	26.9 (9.9–44.0)	0.05
Waywa et al. (2001) [91]	13/288	4.5 (2.1–6.9)	1.88
Wiwanitkit et al. (2001) [89]	3/60	5.0 (0.0–10.5)	0.47
Mohandas et al. (2002) [93]	3/120	2.5 (0.0–5.3)	1.51
Kumar et al. (2002) [92]	14/150	9.3 (4.7–14.0)	0.64
Guk et al. (2005) [95]	5/67	7.5 (1.2–13.8)	0.37
Dwivedi et al. (2007) [48]	2/75	2.7 (0.0–6.3)	0.98
Jayalakshmi et al. (2008) [43]	3/89	3.4 (0.0–7.1)	0.94
Saksirisampant et al. (2009) [101]	1/90	1.1 (0.0–3.3)	2.16
Kulkarni et al. (2009) [44]	11/137	8.0 (3.5–12.6)	0.67
Sherchan et al. (2012) [103]	3/146	2.1 (0.0–4.4)	2.00
Baragundi Mahesh et al. (2013) [106]	7/75	9.3 (2.7–15.9)	0.34
Vyas et al. (2013) [105]	9/75	12.0 (4.6–19.4)	0.27
Mehta et al. (2013) [104]	18/100	18.0 (10.5–25.5)	0.26
Gupta et al. (2013) [45]	25/100	25.0 (16.5–33.5)	0.21
Jain et al. (2014) [108]	2/250	0.8 (0.0–1.9)	4.21
Paboriboune et al. (2014) [107]	6/137	4.4 (1.0–7.8)	1.09
Khalil et al. (2015) [110]	15/200	7.5 (3.8–11.2)	0.98
Kaniyarakkal et al. (2016) [112]	9/200	4.5 (1.6–7.4)	1.44
Mitra et al. (2016) [113]	28/194	14.4 (9.5–19.4)	0.57
Shah et al. (2016) [114]	9/45	20.0 (8.3–31.7)	0.11
Pooled prevalence	215/2848	7.1 (5.2–9.0)	22.21
**Latin America and the Caribbean**			
Escobedo et al. (1999) [116]	1/67	1.5 (0.0–4.4)	1.42
Moran et al. (2005) [147]	1/203	0.5 (0.0–1.5)	4.57
Cardoso et al. (2011) [121]	6/500	1.2 (0.2–2.2)	4.59
Guimarães et al. (2012) [123]	1/93	1.1 (0.0–3.2)	2.26
Assis et al. (2013) [124]	4/59	6.8 (0.4–13.2)	0.35
Pooled prevalence	13/922	1.0 (0.3–1.7)	13.20
**Middle East and North Africa**			
Agholi et al. (2013) [127]	2/356	0.6 (0.0–1.3)	5.06
Salehi Sangani et al. (2016) [128]	2/80	2.5 (0.0–5.9)	1.09
Pooled prevalence	4/436	0.8 (0.0–2.0)	6.15
**Overall pooled prevalence**	**788/105,922**	**2.5 (2.1–2.9)**	**100**

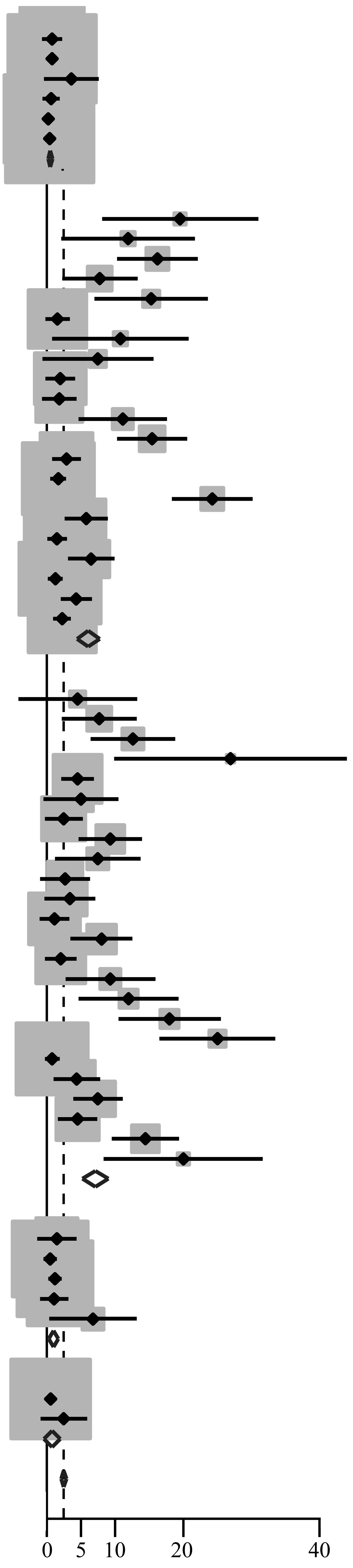

Supplement: Supplementary file 3 — Random-effect meta-analysis of Isospora infection in HIV-infected people. (PDF 345 kb) [file 13071_2017_2558_MOESM3_ESM.pdf]
