# Supplementary material for: Prevalence of Cryptosporidium, microsporidia and Isospora infection in HIV-infected people: a global systematic review and meta-analysis
Source: Parasit Vectors. 2018 Jan 9;11:28. doi: 10.1186/s13071-017-2558-x (PMC5759777; doi:10.1186/s13071-017-2558-x)

Additional file 4: Figure S3. Random-effect meta-analysis of *Isospora* infection in HIV-infected people when the three studies affecting the prevalence of *Isospora* were excluded.

Study ID	Number infected/ total number	Prevalence (%–95% Cl)	% Weight
**Western and central Europe and North America**			
René et al. (1989) [37]	1/132	0.8 (0.0–2.2)	2.72
Mathewson et al.(1998) [42]	3/83	3.6 (0.0–7.6)	1.85
Brandonisio et al. (1999) [38]	1/154	0.6 (0.0–1.9)	2.78
Pooled prevalence	6/572	0.9 (0.0–1.8)	7.35
**Sub-Saharan Africa**			
Henry et al. (1986) [32]	9/46	19.6 (8.1–31.0)	0.50
Colebunders et al. (1988) [56]	5/42	11.9 (2.1–21.7)	0.65
Therizol-Ferly et al. (1989) [57]	24/148	16.2 (10.3–22.2)	1.28
Hunter et al. (1992) [58]	7/90	7.8 (2.2–13.3)	1.38
Dieng et al. (1994) [60]	11/72	15.3 (7.0–23.6)	0.83
Fisseha et al.(1999) [63]	3/190	1.6 (0.0–3.4)	2.64
Lebbad et al.(2001) [65]	4/37	10.8 (0.8–20.8)	0.63
Keshinro et al. (2003) [26]	3/40	7.5 (0.0–15.7)	0.85
Sarfati et al. (2006) [69]	3/154	1.9 (0.0,4.1)	2.50
Mariam et al. (2008) [70]	2/109	1.8 (0.0–4.4)	2.39
Babatunde et al. (2010) [73]	10/90	11.1 (4.6–17.6)	1.15
Alemu et al. (2011) [74]	29/188	15.4 (10.3–20.6)	1.48
Wumba et al. (2012) [144]	7/242	2.9 (0.8–5.0)	2.53
Abaver et al. (2012) [145]	8/480	1.7 (0.5–2.8)	2.81
Nwuba et al. (2012) [33]	49/202	24.3 (18.3–30.2)	1.29
Vouking et al. (2014) [79]	12/207	5.8 (2.6–9.0)	2.14
Girma et al. (2014) [77]	4/268	1.5 (0.0–2.9)	2.73
Bissong et al. (2015) [80]	13/200	6.5 (3.1–9.9)	2.06
Kiros et al. (2015) [81]	5/399	1.3 (0.2–2.3)	2.82
Nsagha et al. (2016) [39]	13/300	4.3 (2.0–6.6)	2.46
Shimelis et al. (2016) [3]	11/491	2.2 (0.9–3.5)	2.77
Pooled prevalence	232/3995	6.1 (4.5–7.7)	37.90
**Asia and the Pacific**			
Punpoowong et al.(1998) [86]	1/22	4.5 (0.0–13.2)	0.78
Wanachiwanawin et al.(1999) [87]	7/91	7.7 (2.2–13.2)	1.40
Mukhopadhya et al. (1999) [146]	14/111	12.6 (6.4–18.8)	1.22
Prasad et al. (2000) [88]	7/26	26.9 (9.9–44.0)	0.25
Waywa et al. (2001) [91]	13/288	4.5 (2.1–6.9)	2.43
Wiwanitkit et al. (2001) [89]	3/60	5.0 (0.0–10.5)	1.39
Mohandas et al. (2002) [93]	3/120	2.5 (0.0–5.3)	2.29
Kumar et al. (2002) [92]	14/150	9.3 (4.7–14.0)	1.64
Guk et al. (2005) [95]	5/67	7.5 (1.2–13.8)	1.20
Dwivedi et al. (2007) [48]	2/75	2.7 (0.0–6.3)	1.98
Jayalakshmi et al. (2008) [43]	3/89	3.4 (0.0–7.1)	1.94
Saksirisampant et al. (2009) [101]	1/90	1.1 (0.0–3.3)	2.51
Kulkarni et al. (2009) [44]	11/137	8.0 (3.5–12.6)	1.67
Sherchan et al. (2012) [103]	3/146	2.1 (0.0–4.4)	2.46
Baragundi Mahesh et al. (2013) [106]	7/75	9.3 (2.7–15.9)	1.13
Vyas et al. (2013) [105]	9/75	12.0 (4.6–19.4)	0.98
Mehta et al. (2013) [104]	18/100	18.0 (10.5–25.5)	0.95
Gupta et al. (2013) [45]	25/100	25.0 (16.5–33.5)	0.81
Jain et al. (2014) [108]	2/250	0.8 (0.0–1.9)	2.82
Paboriboune et al. (2014) [107]	6/137	4.4 (1.0–7.8)	2.05
Khalil et al. (2015) [110]	15/200	7.5 (3.8–11.2)	1.97
Kaniyarakkal et al. (2016) [112]	9/200	4.5 (1.6–7.4)	2.26
Mitra et al. (2016) [113]	28/194	14.4 (9.5–19.4)	1.55
Shah et al. (2016) [114]	9/45	20.0 (8.3–31.7)	0.49
Pooled prevalence	215/2848	7.1 (5.2–9.0)	38.16
**Latin America and the Caribbean**			
Escobedo et al. (1999) [116]	1/67	1.5 (0.0–4.4)	2.25
Moran et al. (2005) [147]	1/203	0.5 (0.0–1.5)	2.85
Cardoso et al. (2011) [121]	6/500	1.2 (0.2–2.2)	2.85
Guimarães et al. (2012) [123]	1/93	1.1 (0.0–3.2)	2.53
Assis et al. (2013) [124]	4/59	6.8 (0.4–13.2)	1.17
Pooled prevalence	12/719	1.3 (0.5–2.1)	11.65
**Middle East and North Africa**			
Agholi et al. (2013) [127]	2/356	0.6 (0.0–1.3)	2.88
Salehi Sangani et al. (2016) [128]	2/80	2.5 (0.0–5.9)	2.06
Pooled prevalence	4/436	0.8 (0.0–2.0)	4.94
**Overall pooled prevalence**	**469/8570**	**5.0 (4.1–5.9)**	**100.00**

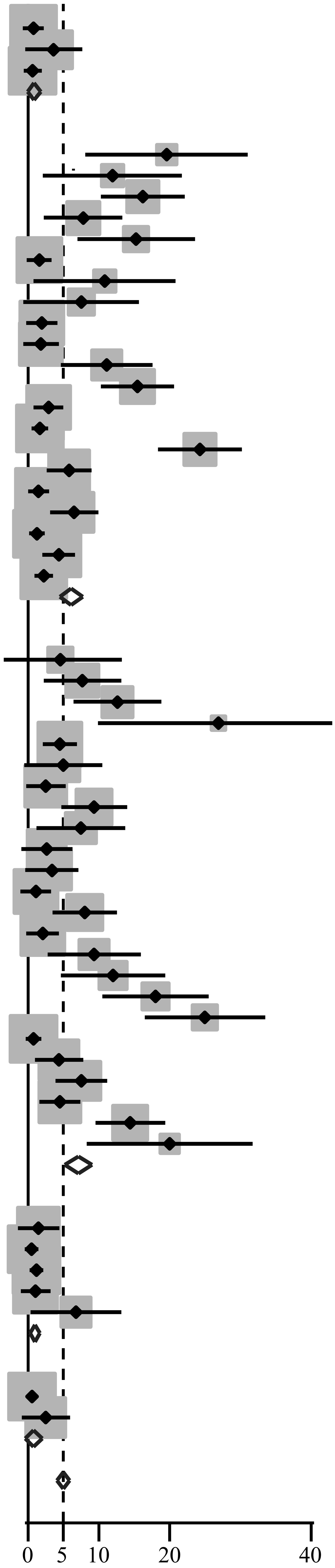

Supplement: Supplementary file 4 — Random-effect meta-analysis of Isospora infection in HIV-infected people when the three studies affecting the prevalence of Isospora were excluded. (PDF 344 kb) [file 13071_2017_2558_MOESM4_ESM.pdf]
