# Supplementary material for: Prevalence of Cryptosporidium, microsporidia and Isospora infection in HIV-infected people: a global systematic review and meta-analysis
Source: Parasit Vectors. 2018 Jan 9;11:28. doi: 10.1186/s13071-017-2558-x (PMC5759777; doi:10.1186/s13071-017-2558-x)

Additional file 5: Figure S4. Random-effect meta-analysis of the association of diarrhea with *Cryptosporidium* infection in HIV-infected people.

Study ID	Number infected/total number: Diarrhea	Number infected/total number: Control	Odds ratio (95% CI)	% Weight
Hunter et al. (1992) [58]	2/59	0/31	2.74 (0.13–58.85)	2.21
Anand et al. (1996) [85]	70/150	0/50	88.45 (5.36–1460.10)	2.47
Fisseha et al. (1999) [63]	38/147	0/43	30.59 (1.84–508.99)	2.46
Brandonisio et al. (1999) [38]	14/65	3/89	7.87 (2.16–28.71)	4.71
Wiwanitkit et al. (2001) [89]	2/28	0/32	6.13 (0.28–133.34)	2.19
Sarfati et al. (2006) [69]	3/46	3/108	2.44 (0.47–12.58)	4.10
Dwivedi (2007) [48]	23/50	2/25	9.80 (2.08–46.07)	4.26
Ramakrishnan et al. (2007) [97]	23/80	3/80	10.36 (2.97–36.18)	4.79
Goncalves et al. (2009) [120]	6/40	3/60	3.35 (0.79–14.29)	4.43
Cardoso et al. (2011) [121]	0/143	1/357	0.83 (0.03–20.45)	2.08
Velasco et al. (2011) [122]	3/16	13/115	1.81 (0.46–7.21)	4.55
Wumba et al. (2012) [76]	7/83	6/159	2.35 (0.76–7.23)	5.01
Sherchan et al. (2012) [103]	4/78	0/68	8.28 (0.44–156.55)	2.33
Wang et al. (2013) [9]	3/263	7/328	0.53 (0.14–2.07)	4.59
Assis et al. (2013) [124]	5/23	2/36	4.72 (0.83–26.81)	3.93
Gupta et al. (2013) [45]	4/19	0/81	47.32 (2.42–924.06)	2.30
Girma et al. (2014) [77]	88/242	4/26	3.14 (1.05–9.41)	5.06
Paboriboune et al. (2014) [107]	5/59	4/78	1.71 (0.44–6.68)	4.59
Samie et al. (2014) [78]	24/97	16/54	0.78 (0.37–1.64)	5.64
Jain et al. (2014) [108]	52/76	0/174	747.86 (44.72–12,507.70)	2.45
Bissong et al. (2015) [80]	13/77	1/123	24.78 (3.17–193.72)	3.43
Khalil et al. (2015) [110]	12/100	3/100	4.41 (1.20–16.14)	4.70
Mitra et al. (2016) [113]	26/74	31/120	1.56 (0.83–2.92)	5.80
Shimelis et al. (2016) [3]	38/311	27/180	0.79 (0.46–1.34)	5.92
Shah et al. (2016) [114]	4/27	2/18	1.39 (0.23–8.53)	3.81
Wang et al. (2016) [18]	2/30	0/255	44.83 (2.10–956.89)	2.21
**Overall**	**471/2383**	**131/2790**	**4.09 (2.32–7.20)**	**100.00**

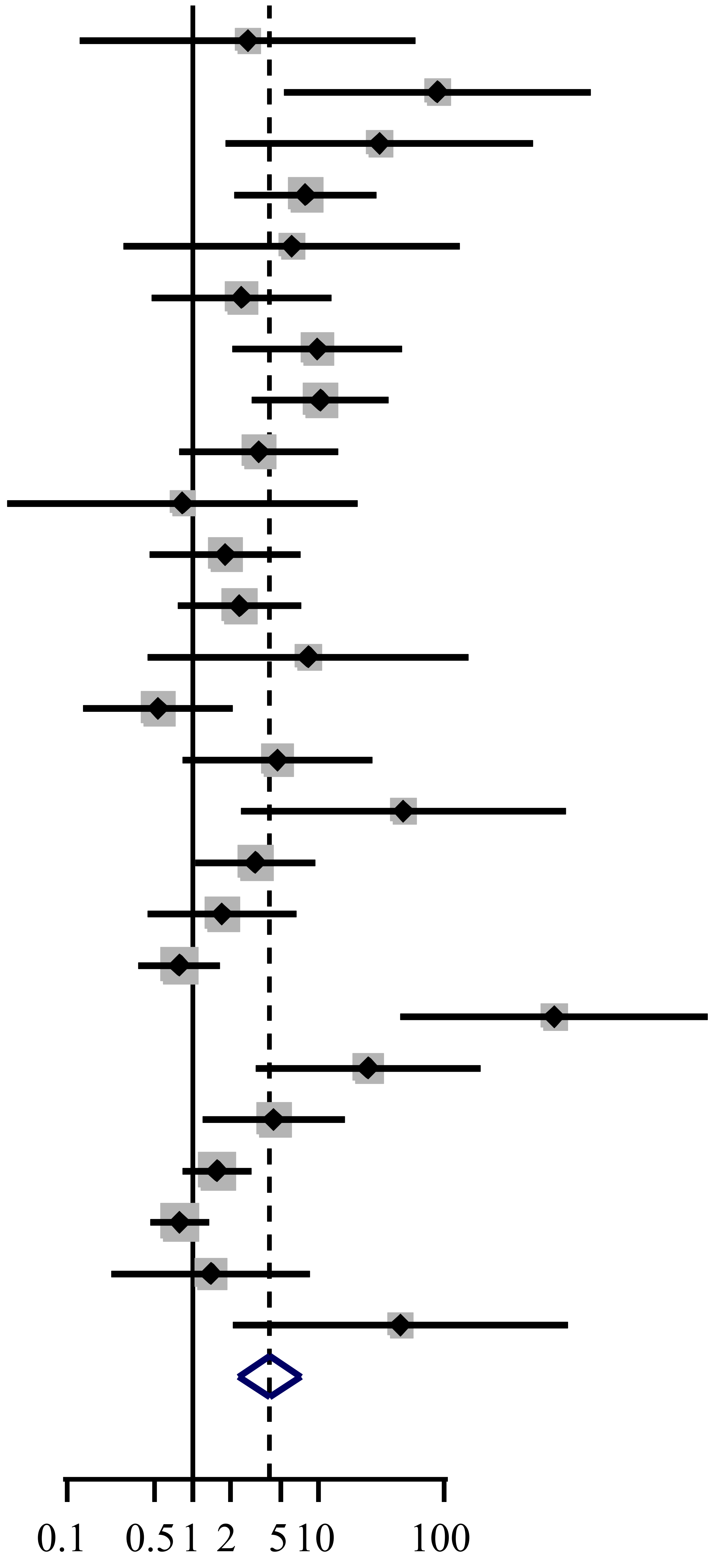

Supplement: Supplementary file 5 — Random-effect meta-analysis of the association of diarrhea with Cryptosporidium infection in HIV-infected people. (PDF 227 kb) [file 13071_2017_2558_MOESM5_ESM.pdf]
