# Supplementary material for: Prevalence of Cryptosporidium, microsporidia and Isospora infection in HIV-infected people: a global systematic review and meta-analysis
Source: Parasit Vectors. 2018 Jan 9;11:28. doi: 10.1186/s13071-017-2558-x (PMC5759777; doi:10.1186/s13071-017-2558-x)

Additional file 6: Figure S5. Fixed-effect meta-analysis of the association of diarrhea with microsporidian infection in HIV-infected people.

Study ID	Diarrhea (Number infected/total number)	Control (Number infected/total number)	Odds ratio (95% CI)	% Weight
Weber et al. (1992) [131]	6/27	0/107	65.00 (3.53–1197.34)	0.39
Kotler et al. (1994) [132]	55/141	1/53	33.26 (4.47–247.57)	2.20
Coyle et al. (1996) [133]	30/68	1/43	33.16 (4.31–255.03)	1.70
Brandonisio et al. (1999) [38]	6/65	1/89	8.95 (1.05–76.26)	1.90
Wiwanitkit et al. (2001) [89]	1/28	0/32	3.55 (0.14–90.59)	1.10
Kumar et al. (2002) [92]	1/100	0/50	1.52 (0.06–38.05)	1.63
Chacin-Bonilla et al. (2006) [119]	13/74	1/29	5.97 (0.74–47.89)	2.94
Sarfati et al. (2006) [69]	3/46	5/108	1.44 (0.33–6.28)	6.94
Dwivedi et al. (2007) [48]	5/50	0/25	6.17 (0.33–116.08)	1.47
Akinbo et al. (2012) [139]	26/98	52/365	2.17 (1.27–3.72)	40.19
Wumba et al. (2012) [76]	12/83	8/159	3.19 (1.25–8.15)	11.67
Agholi et al. (2013) [127]	8/103	0/253	45.13 (2.58–789.43)	0.66
Wang et al. (2013) [9]	23/263	12/328	2.52 (1.23–5.17)	24.22
Bissong et al. (2015) [80]	4/77	0/123	15.12 (0.80–284.91)	0.90
Khalil et al. (2015) [110]	5/100	0/100	11.58 (0.63–212.19)	1.17
Mitra et al. (2016) [113]	4/74	0/120	15.38 (0.82–289.96)	0.89
**Overall**	**202/1397**	**81/1984**	**4.72 (3.47–6.42)**	**100.00**

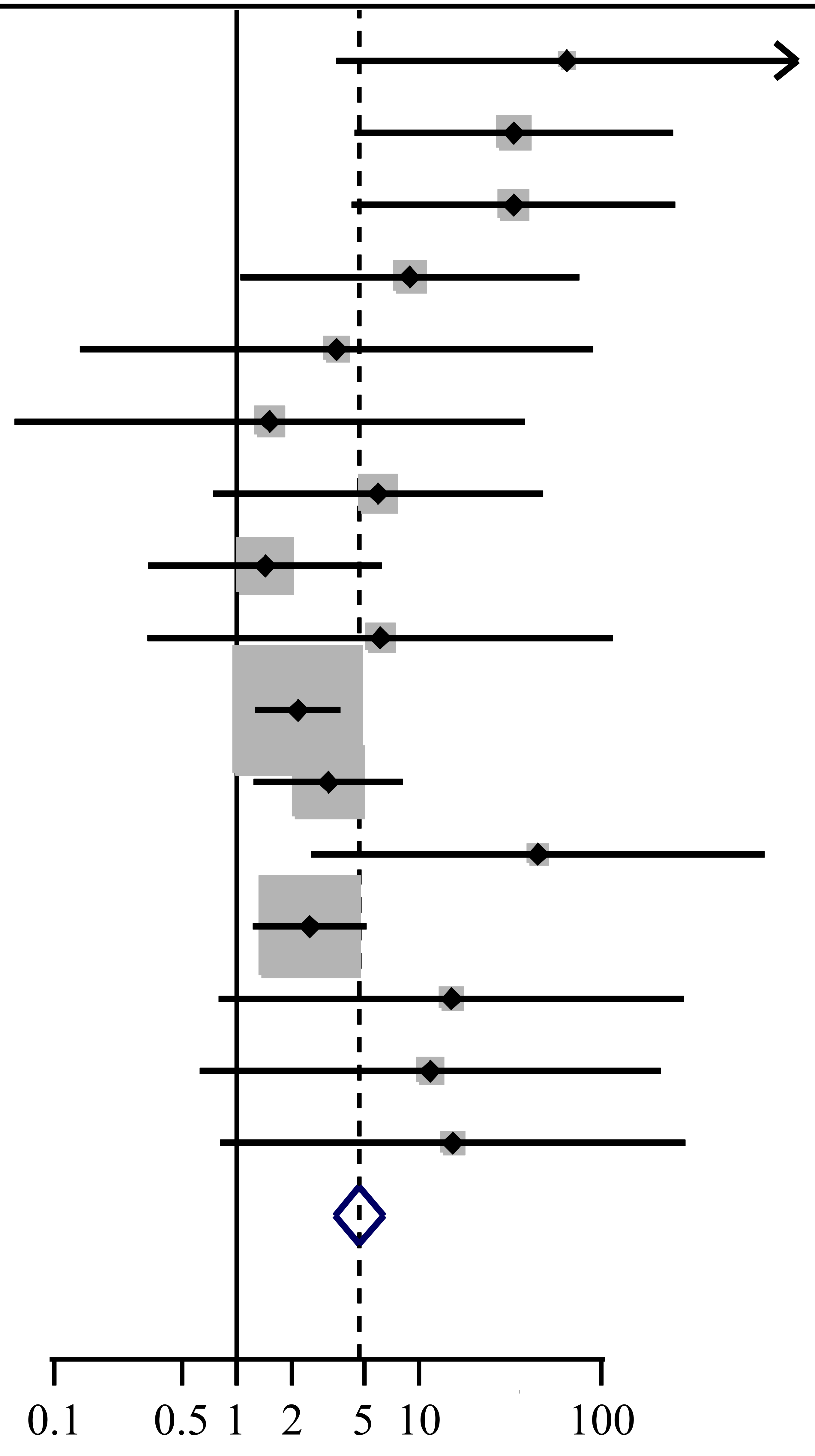

Supplement: Supplementary file 6 — Fixed-effect meta-analysis of the association of diarrhea with microsporidia infection in HIV-infected people. (PDF 170 kb) [file 13071_2017_2558_MOESM6_ESM.pdf]
