# Supplementary material for: Prevalence of Cryptosporidium, microsporidia and Isospora infection in HIV-infected people: a global systematic review and meta-analysis
Source: Parasit Vectors. 2018 Jan 9;11:28. doi: 10.1186/s13071-017-2558-x (PMC5759777; doi:10.1186/s13071-017-2558-x)

Additional file 7: Figure S6. Fixed-effect meta-analysis of the association of diarrhea with *Isospora* infection in HIV-infected people.

Study ID	Number infected/total number: Diarrhea	Number infected/total number: Control	Odds ratio (95% CI)	% Weight
Hunter et al. (1992) [58]	7/59	0/31	9.00 (0.50–163.01)	2.14
Fisseha et al. (1999) [63]	2/147	1/43	0.58 (0.05–6.55)	5.73
Brandonisio et al. (1999) [38]	1/68	0/89	4.16 (0.17,103.83)	1.55
Mukhopadhya et al. (1999) [146]	11/61	2/50	5.28 (1.11–25.07)	6.77
Wiwanitkit et al. (2001) [89]	2/28	1/32	2.39 (0.20–27.81)	3.26
Kumar et al. (2002) [92]	14/100	0/50	16.93 (0.99–289.92)	2.14
Sarfati et al. (2006) [69]	3/46	0/108	17.46 (0.88–345.12)	1.05
Dwivedi et al. (2007) [48]	2/50	0/25	2.63 (0.12–56.86)	2.37
Cardoso et al. (2011) [121]	6/143	0/357	33.80 (1.89–604.03)	1.03
Abaver et al. (2012) [145]	5/176	3/304	2.93 (0.69–12.43)	8.03
Sherchan et al. (2012) [103]	3/78	0/68	6.35 (0.32–125.19)	1.92
Assis et al. (2013) [124]	2/23	2/36	1.62 (0.21–12.38)	5.35
Gupta et al. (2013) [45]	9/19	1/81	72.00 (8.24–629.37)	0.75
Jain et al. (2014) [108]	2/76	0/174	11.71 (0.56–246.91)	1.11
Girma et al. (2014) [77]	4/242	0/26	1.00 (0.05–19.09)	3.32
Paboriboune et al. (2014) [107]	3/59	3/78	1.34 (0.26–6.89)	9.21
Bissong et al. (2015) [80]	8/77	5/123	2.74 (0.86–8.70)	12.96
Khalil et al. (2015) [110]	10/100	5/100	2.11 (0.70–6.42)	16.90
Mitra et al. (2016) [113]	24/74	4/120	13.92 (4.59–42.21)	7.74
Shah et al. (2016) [114]	7/27	2/18	2.80 (0.51–15.38)	6.68
**Overall**	**125/1,650**	**29/1,913**	**4.93 (3.33–7.29)**	**100.00**

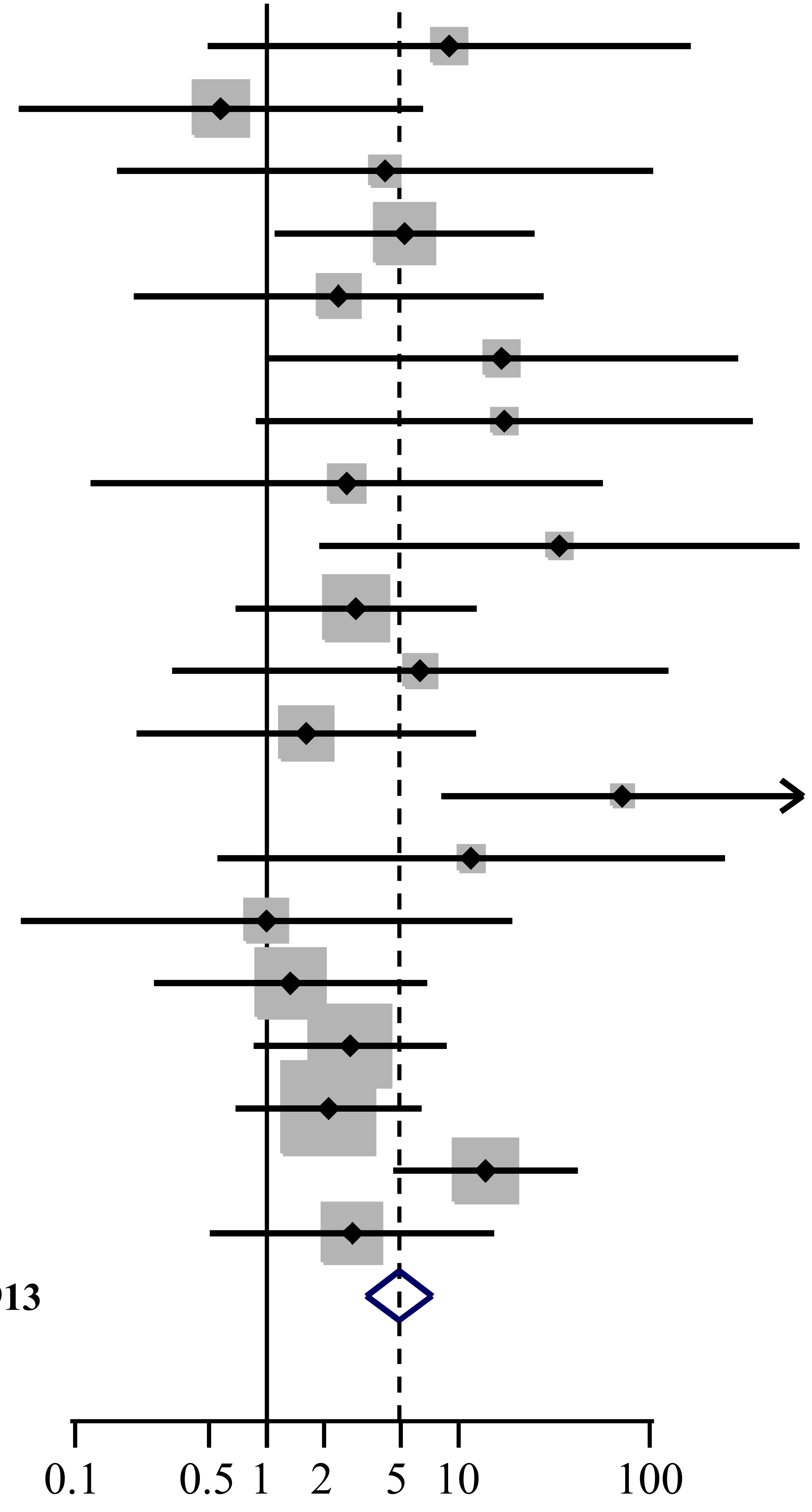

Supplement: Supplementary file 7 — Fixed-effect meta-analysis of the association of diarrhea with Isospora infection in HIV-infected people. (PDF 217 kb) [file 13071_2017_2558_MOESM7_ESM.pdf]
